# Cardiotoxicity adverse outcome pathway network: towards mechanistic and quantitative modelling

**DOI:** 10.3389/ftox.2026.1781536

**Published:** 2026-05-18

**Authors:** Luiz Ladeira, Devon A. Barnes, Rosalinde Masereeuw, Liesbet Geris, Bernard Staumont

**Affiliations:** 1 Biomechanics Research Unit, GIGA Institute, University of Liège, Liège, Belgium; 2 Division of Pharmacology, Utrecht Institute for Pharmaceutical Sciences, Utrecht University, Utrecht, Netherlands; 3 Skeletal Biology and Engineering Research Center, KU Leuven, Leuven, Belgium; 4 Biomechanics Section, Department of Mechanical Engineering, KU Leuven, Leuven, Belgium

**Keywords:** adverse outcome pathways, cardiac safety, next-generation risk assessment, systems biology, toxicity

## Abstract

**Introduction:**

Chemical-induced heart toxicity remains a major challenge in drug development and environmental safety, largely because current testing often relies on narrow, late-stage endpoints that miss the complex biological progression of the toxicities. To address this, we developed a comprehensive Adverse Outcome Pathway (AOP) network that maps how early (bio) chemical triggers evolve into organ-level dysfunction.

**Methods:**

By integrating data from the OECD AOP-Wiki, we constructed a unified network of 64 biological events and 94 documented relationships that identifies the critical biological “crossroads” where different toxic chemicals converge to cause heart damage.

**Results/discussion:**

Our analysis reveals a compact core of central biological events, such as oxidative stress and mitochondrial dysfunction, which act as the primary drivers of cardiac injury. This network approach moves beyond single, linear pathways to show how systemic factors, including interactions with other organs like the kidneys, contribute to cardiotoxicity. To translate these findings into a practical resource for the broader scientific community, we developed a methods catalogue that links these biological events to specific laboratory assays. To ensure this work is accessible and actionable, we hosted the network on an interactive, FAIRaligned web platform. By providing a clear scaffold for understanding heart safety, this resource enables the design of more human-relevant, animal-free testing strategies and helps prioritise the most impactful biomarkers for future safety assessments.

## Plain language summary

Why this study matters: Determining if a chemical or drug will damage the heart is a major challenge in safety testing and impacts drug development. Traditionally, researchers look at the final stages of heart damage, often missing the early warning signs. In this study, we created a digital map that shows the “chain reaction” of events, starting from a chemical interaction in the body and leading all the way to heart failure.

What we did: By gathering data from a global scientific database, we built a network of 64 biological events that lead to heart toxicity. Our map identifies “biological crossroads”, which are specific moments like cell stress or power failures within the cell, where many different toxic chemicals converge to cause damage. We also describe how other organs, like the kidneys, play an important role in how the heart reacts to these chemicals.

The impact: We made this map available in an easy-to-use website and provided a catalogue of testing methods. This resource helps scientists design new, animal-free tests by focusing on the most important biological signs of danger. Ultimately, this can potentially make drug and other chemical developments safer by catching heart risks much earlier than before.

## 1 Introduction

Cardiovascular diseases remain the leading cause of mortality worldwide (Global Burden of Cardiovascular Diseases and Risks, 2023 Collaborators, 2025; OECD, 2025), with chemical-induced cardiotoxicity representing a significant and growing concern across both therapeutic and environmental contexts (Daley et al., 2022; Georgiadis et al., 2022). Mechanistically, cardiotoxicity arises from a diverse range of perturbations, including mitochondrial dysfunction, oxidative stress, calcium mishandling, inflammation, and disruption of ion homeostasis, ultimately leading to cardiac muscle damage. These processes are not only biologically complex but also temporally variable, with adverse outcomes manifesting over timescales ranging from seconds to decades (Mamoshina et al., 2021). This variability poses a significant challenge for traditional detection methods, primarily focusing on functional endpoints, such as contractility and hERG inhibition, which often fail to capture the full spectrum of cardiotoxic effects (Daley et al., 2022). Such limitation contributes to high attrition rates in pharmaceutical development and persistent gaps in evaluating environmental chemicals (Daley et al., 2022; Mamoshina et al., 2021).

Beyond the pharmaceutical context, environmental cardiotoxicity has gained increasing attention as a public health concern, with mounting evidence linking cardiovascular dysfunction to chronic exposure to pollutants such as airborne particulates, pesticides, and endocrine-disrupting chemicals (Daley et al., 2022; Georgiadis et al., 2022; Schaffert et al., 2023). Despite this, cardiotoxicity remains unclassified as an independent hazard category in regulatory frameworks, limiting recognition and hindering the development of tailored assessment strategies (Georgiadis et al., 2022). Epidemiological data suggest that 7%–23% of the cardiovascular disease burden may be attributable to environmental exposures, yet the underlying toxicological mechanisms remain insufficiently characterised (Daley et al., 2022). Modern *in vitro* and *in silico* models, while promising, often focus on a limited spectrum of, however relevant, targets, such as ion channels, failing to capture the broader mechanistic landscape necessary for understanding cardiotoxicity (Daley et al., 2022). This systemic lack of mechanistic understanding underscores the urgent need for more comprehensive, mechanism-based approaches (Bajard et al., 2023) bridging effective protective strategies and regulatory frameworks for both pharmaceutical and environmental chemical safety.

To address these challenges, mechanism-based frameworks are needed to complement functional screening and enhance predictive resolution. The Adverse Outcome Pathway (AOP) framework offers a conceptual construct designed to transform toxicity testing and accelerate evidence-based risk assessment by leveraging data from multiple sources. An AOP is defined as a sequence of measurable key events (KE), starting from a molecular initiating event (MIE) and leading towards an adverse outcome (AO), connected via key event relationships (KERs), which provide the scientific foundation for causal inferences (Ankley et al., 2009; OECD, 2018; Vinken et al., 2017). It provides a structured, stressor-agnostic way to organise existing mechanistic knowledge (Vinken et al., 2017). AOPs delineate causal sequences of disturbed events across molecular, cellular, tissue, and organ scales that ultimately produce adverse health or ecotoxicological outcomes at the individual or population level (Ankley et al., 2009). Their modular architecture enables reuse and recombination of KEs across pathways, supporting both knowledge integration and systematic application. Although AOPs are typically developed as linear constructs, combining them into networks better reflects the complexity of biological systems (Knapen et al., 2018; Villeneuve et al., 2014) which makes them especially valuable for capturing multiscale mechanisms such as multi-organ cross-talks.

The AOP-Wiki, developed and maintained under the oversight of the Organisation for Economic Cooperation and Development (OECD), constitutes the primary public platform for AOP development and dissemination (OECD, 2017; OECD, 2018). It structures mechanistic knowledge grounded in expert curation and community contributions. Recent assessments have revealed notable gaps and imbalances in the coverage of biological domains, with cardiovascular mechanisms under-represented despite their regulatory relevance (Jaylet et al., 2024). In particular, KEs such as oxidative stress and related processes, central to cardiotoxicity, are inconsistently annotated or insufficiently linked to downstream outcomes. Recent efforts have addressed these gaps by promoting harmonised KE terminology, advancing data-driven mapping approaches (Mortensen et al., 2022; Mortensen et al., 2025; Tanabe et al., 2022; Tanabe et al., 2023; Wittwehr et al., 2023; Wittwehr et al., 2025).

Extending this approach to the heart requires adapting established network-based methodologies to the specific mechanistic landscape of cardiotoxicity. The cardiotoxicity-focused network presented in this work builds on the modular structure of the AOP-Wiki to integrate dispersed information into a coherent and biologically relevant network. To our knowledge, this is the first network-level integration of AOP-Wiki resources, specifically focused on cardiotoxicity, providing a systematic map of how chemical-induced events propagate to cardiac outcomes. The resulting representation facilitates the identification of recurrent KEs across different contexts, highlighting shared mechanisms and systemic contributors, such as cardio-renal interactions. These cross-cutting events are not merely descriptive, as they are prioritised through network topological metrics to inform assay selection, identify mechanistic gaps, and support the development of quantitative models for predictive toxicology. This resource therefore represents both a conceptual and operational advance, offering a foundational scaffold for integrating existing data, designing test batteries, and anchoring new methods to clinically relevant mechanisms. Prior applications in liver, kidney, endocrine and nervous system toxicology have demonstrated the utility of this strategy in identifying mechanistically anchored, measurable and biologically relevant KEs (Arnesdotter et al., 2021; Barnes et al., 2024; Spinu et al., 2019; Wiklund et al., 2023). In this study, we apply a transparent screening and curation workflow to the AOP-Wiki to assemble and characterise an AOP network for cardiotoxicity, with the goal of identifying relevant KEs as a foundation for future animal-free and human-relevant quantitative *in vitro* and *in silico* toxicity predictions.

## 2 Methods

We built a data-driven AOP network following already established methods (Arnesdotter et al., 2021; Barnes et al., 2024; Spinu et al., 2019; Wiklund et al., 2023). Briefly, it includes definition of purpose, data collection and screening process, inclusion and exclusion criteria, data curation and harmonisation, and network building, followed by a comprehensive analysis as previously described (Villeneuve et al., 2014). We also mapped the KE quantification methods reported at AOP-Wiki and built an interactive catalogue that can be displayed in an interactive version of the network, as described in the sections below.

### 2.1 Definition of purpose

This study aimed to identify, describe, and investigate the mechanisms of cardiotoxicity caused by chemical stimuli previously documented in the AOP-Wiki database. AOPs describing cardiotoxicity and the KEs associated with cardiotoxic mechanisms were integrated into an AOP network and analysed to better understand the relationships between MIEs and adverse outcomes (AO) in the heart.

### 2.2 Data collection and screening process

A manual search of the AOP-Wiki (https://aopwiki.org/) was conducted on 12 August 2025, to identify individual AOPs concerning cardiotoxicity. The Key Events dataset was downloaded (https://aopwiki.org/downloads/aop_ke_mie_ao.tsv) and searched for KEs and AOs associated with cardiotoxicity using the following keywords: “heart”, “cardio”, “cardiac”, “ventricular”, “ventricle”, “myocardial”, “myocardium”, “atrial”, “atrio”, and “atrium” (Supplementary Data S1).

We intentionally excluded vascular-related keywords from the KEs screening because this study focused specifically on toxicological mechanisms primarily affecting cardiac tissue and function. While purely vascular mechanisms fall outside our primary search scope, cardiac and vascular dysfunctions are frequently tightly linked. To handle this, we employed a strict pathway-level criterion, if an AOP contained at least one KE or AO with direct mechanistic link to the heart (as established by the keywords used), the entirety of the AOP entry was considered for inclusion. As is evident in the results, some vascular-related or other non-myocardium-related KEs are present in the final network. This approach ensures the capture of cross-organ mechanisms while remaining grounded to a cardiac event based on the inclusion/exclusion criteria.

This initial search identified 19 AOPs containing KEs related to cardiotoxicity: AOP:16, AOP:21, AOP:94, AOP:104, AOP:138, AOP:150, AOP:177, AOP:186, AOP:261, AOP:304, AOP:377, AOP:426, AOP:427, AOP:433, AOP:436, AOP:456, AOP:479, AOP:480, and AOP:539. A subsequent manual search for AOP titles using the same keywords directly on the database identified three additional AOPs: AOP:438, AOP:448, and AOP:515.

Although we adopted a systematic approach for screening, and data-driven programmatic method in order to enhance reproducibility, it is important to state that there was no independent processing and cross-comparison of outcomes, which usually is employed in systematic reviews for instance. Outcomes were discussed and validated by all authors, and all the included and excluded elements and data visualisation were cross-checked by domain experts.

### 2.3 Inclusion and exclusion criteria

AOPs were assessed and included if they contained at least one key event (KE) or AO explicitly related to cardiotoxicity. Once an AOP met this cardiac inclusion criterion, all KEs within that specific pathway were retained to preserve mechanistic continuity. On the other hand, AOPs were excluded based on the following criteria:

- Lack of KER information (n = 3): AOP:186, AOP:377, AOP:448.
- All rights reserved and no permission granted (n = 2): AOP:438, AOP:515.
- COVID-19-related (n = 2): AOP:426, AOP:427.
- Vasculature and development-related (n = 2): AOP:304, AOP:436.
- Fish-specific (n = 1): AOP:539.

Although other AOPs also include fish in their taxonomy domain, AOP:539 was the only one among them specifically designed for freshwater fish species, including the “KE2236 - Decreased, Sodium uptake in gills”, which led to its exclusion from the analysis. At the time of this publication, no reuse permission was obtained from the AOPs with an “all rights reserved” licence, leading to exclusion from the analysis.

**FIGURE 1:**
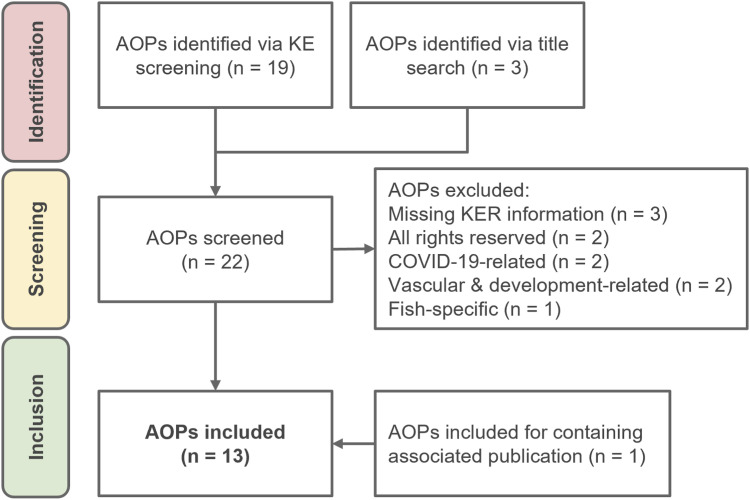
AOP-Wiki screening process for cardiotoxicity-related Adverse Outcome Pathways.

Figure 1 illustrates the screening process. One AOP (AOP:448) was initially excluded due to lack of KERs information in the AOP-Wiki database. However, a publication regarding this AOP was identified, and it was subsequently re-included in the analysis. Supplementary Data S2 contains an overview of the AOPs screened.

### 2.4 Key event harmonisation

To ensure consistency in the network analysis, Key Event (KE) titles with similar biological meanings or functions were merged. Duplicated KEs were identified and grouped under common terms. In addition, we standardised nomenclature with uppercase letters at the beginning of the names and removed NA entries. The complete harmonised data can be found in Supplementary Data S3.

### 2.5 Network building and visualization

Next, AOP data was extracted manually from each entry and/or their related publications. The data was consolidated in a spreadsheet and cleaned by keeping only the columns necessary for data visualization and analysis (Supplementary Data S4, S5), which was used for the network building and subsequent analysis.

**FIGURE 2:**
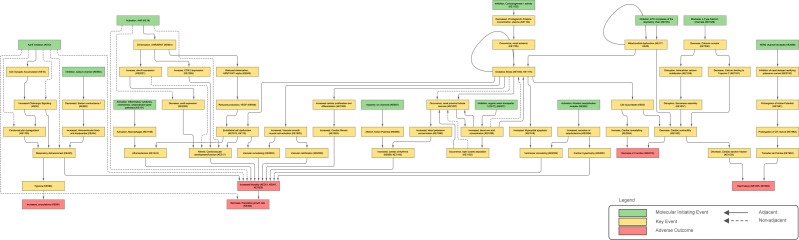
Adverse Outcome Pathway network of 13 AOPs of cardiotoxicity available on the AOP-Wiki as of 12 August 2025.

The AOP network was built using the open-source software platform Cytoscape (v. 3.10.4; https://cytoscape.org/) (Shannon et al., 2003). The network was automatically organised using the yFiles Hierarchic Layout algorithm. Additional annotations, including KE adjacency (edge type – dotted or continuous) and type (node colour), were added to enhance the clarity of the network’s visual components. Supplementary Data S6 contains node attributes and was used to support the visualisation generation. KEs were represented as nodes in the network and classified as MIEs (green), KEs (yellow), or AOs (red). Adjacent Key Event Relationships (KERs) were represented as directed continuous edges (connecting arrows), and non-adjacent KERs were represented as directed dotted edges, linking upstream and downstream KEs. The final network representation is shown in Figure 2.

### 2.6 Network analysis

The distribution of node types was quantified to assess the proportional representation of event types in the cardiotoxicity network. Node relationships were categorised according to their adjacency (adjacent vs. non-adjacent) and the level of supporting evidence (high, moderate, or unspecified). Evidence levels and the degree of quantitative understanding for each relationship were extracted from AOP-Wiki entries and categorised accordingly. For analysis purposes, evidence levels labelled as “High” or “Strong” were grouped under “High,” while “Not Specified” and “NA” were consolidated under “Not Specified.”

The Cytoscape Network Analyzer tool was used to compute standard network metrics considering a directed network. Several network metrics were calculated to identify critical nodes. The betweenness centrality was calculated for each node to measure its importance as a bridge connecting different parts of the network. Total degree (sum of in-degree and out-degree) was calculated for each node to identify the most connected nodes in the network. Stress centrality was calculated to identify critical convergence hubs, measuring the total number of shortest paths passing through a node. A combined importance score was computed by normalising and summing the above centrality measures to identify the overall most critical nodes in the network.

Nodes were classified as convergent or divergent based on their connectivity patterns. Convergent nodes were considered those with more incoming connections than outgoing (in-degree > out-degree). On the contrary, divergent nodes are those with more outgoing connections than incoming (out-degree > in-degree). This classification helped identify key regulatory points and potential intervention targets within the cardiotoxicity network.

Overall network metric results can be found in Supplementary Data S7. A reproducible R script for this analysis is provided in Supplementary Scripts.

The Pesca3.0 Cytoscape plugin (https://apps.cytoscape.org/apps/pesca30) was used to compute the simple path occurrence (Scardoni et al., 2015), which represents a more appropriate metric for betweenness centrality in the AOP network context (Barnes et al., 2024). The distribution of path lengths within the network was analysed to assess network compactness and efficiency in information flow. For each KE, simple path occurrence was defined as the number of distinct MIE to AO simple paths in which the KE appears as an internal node. This was visualised as a histogram showing the frequency of paths of different lengths. Simple path metrics can be found in Supplementary Data S8.

### 2.7 Mapping KEs to key characteristics (KCs) of cardiovascular toxicants

To enhance context-specific interpretation of the network and its components, KEs where mapped to established key characteristics of cardiovascular toxicants (Lind et al., 2021). This was done by interpreting the definition of each KC as described in the original publication and relating them to each KE description. Results are provided in the Supplementary Table S1.

### 2.8 Cataloguing methods for KE detection

A catalogue of detection methods was built by collecting and organising information available at each KE unique page in AOP-Wiki, especially in the section “How It Is Measured or Detected”. The section text from each KE was automatically retrieved from the latest release of the AOP-Wiki XML database (version 2025-07-01) available at https://aopwiki.org/downloads/aop-wiki-xml-2025-07-01.gz. We extracted the text for all KEs present in our network (script provided in Supplementary Scripts), organised, checked manually AOP-Wiki entries for missing data and summarised the information available in a catalogue, which is available as Supplementary Data S9.

### 2.9 Interactive visualization of the AOP network and related data

Following the example of Mazein et al. (2025), we prepared an online interactive version of the AOP network for user exploration and for network interoperability, reuse and sharing. For this, an Systems Biology Markup Language (SBML) representation of the AOP network was generated using a dedicated R script based on the minervar package (Gawron et al., 2023). To this end, the network data were pre-processed, annotated, and converted into a CellDesigner-compatible SBML format (Funahashi et al., 2008). Due to CellDesigner constraints, the two self-loops present in the network could not be represented in this interactive version. The network layout was then manually curated within CellDesigner. Context annotations (e.g., biological level and organ- or cell-type where the KE happens) were assigned to KEs in order to organise the network in a context-specific manner, visualising KEs on top of the cells, organs, compartments, or human-scale level. The context annotations can be found in Supplementary Data S6. The final model was deployed on the MINERVA (Molecular Interaction NEtwoRk VisuAlization) platform (Hoksza et al., 2020) for interactive exploration and is accessible online at https://cardiotox.elixir-luxembourg.org/.

To illustrate evidence levels and the degree of quantitative understanding for each KER, annotations from AOP-Wiki were processed and visualised as overlays within MINERVA. A reproducible R script for handling categorical data overlay is provided in Supplementary Scripts. This script formats data according to MINERVA’s overlay specifications, including standardised headers with version information, descriptive labels, and descriptions. Output files were exported as tab-separated text files compatible with MINERVA’s overlay visualisation engine.

It is important to note that different data types may require tailored processing to optimise visualisation within the MINERVA environment. Users are encouraged to consult the MINERVA User Manual at https://minerva.pages.uni.lu/doc/ for detailed guidance on creating fit-for-purpose overlays.

Moreover, a MINERVA plugin (Hoksza et al., 2019) was prepared to dynamically map KE detection methods to the displayed network. For that, a JavaScript plugin was designed to access and display information from a Google Spreadsheet, map the information against the nodes in the network and tag the nodes dynamically displayed in the plugin table. The data source used is the catalogue of methods for KE detection built from AOP-Wiki entries. The plugin source code can be accessed via https://github.com/luiz-ladeira/cardiotox_aop_minerva_plugin and the plugin can be used in the MINERVA platform by loading it with the following link: https://raw.githubusercontent.com/luiz-ladeira/cardiotox_aop_minerva_plugin/master/plugin.js.

### 2.10 Software

All the analyses were done using R version 4.4.0 (2024-04-24 ucrt) (R Core Team, 2024) and R Studio version 2023.12.1.402 (Ocean Storm) (Posit team, 2024). Visualisation of the network was generated using Cytoscape version 3.10.4 (Shannon et al., 2003) and graphs were generated using ggplot2 (Wickham, 2016). The network layout was curated in CellDesigner version 4.4 (Funahashi et al., 2008). Interactive visualisation is provided using the MINERVA platform version 20 (Hoksza et al., 2019). All scripts are provided in Supplementary Scripts.

## 3 Results

### 3.1 Assembling the cardiotoxicity AOP network

A total of 22 linear AOPs were initially identified through a manual AOP-Wiki search and title screening (Figure 1). After applying the predefined inclusion and exclusion criteria, 13 AOPs were retained for inclusion in the cardiotoxicity network (Supplementary Data S2). These selected AOPs reflect a diverse array of initiating mechanisms and AOs relevant to cardiac toxicity. MIEs included acetylcholinesterase inhibition (AOP:16), aryl hydrocarbon receptor (AhR) activation (AOPs:21, 150, 456), ion channel perturbations such as sodium channel inhibition and hERG channel blockade (AOPs:94, 104, 433), and inhibition of mitochondrial electron transport chain complexes (AOPs:479, 480). The resulting AOs ranged from non-specific endpoints like increased mortality to more organ-specific manifestations such as decreased left ventricular function, heart failure, and sudden cardiac death. Two AOPs (AOPs:21, 150) have received OECD endorsement, indicating that they have completed the OECD AOP Programme’s peer-review and quality-control process. One was under review (AOP: 456), while the remaining are still under development (30.76%) or have not yet undergone formal evaluation (46.15%).

Although AOP:433 targets “sudden cardiac death” in AOP-Wiki, during KE harmonisation this outcome was consolidated under “Heart failure (KE1535, KE 1964)” to avoid duplication and maintain a compact AO layer (Supplementary Data S3). AOP:448 was included despite lacking formally defined MIE and AO annotations in the AOP-Wiki, as its structure and mechanistic components are comprehensively described in an associated peer-reviewed publication (Ding et al., 2022). Similarly, additional AOPs included in the network are supported by published literature that complements or expands upon the information available in the AOP-Wiki. For instance, the current status of AOP:16 on AOP-Wiki is “under development” but has its mechanistic basis and key relationships detailed in a published article (Russom et al., 2014), while AOPs:21 and 150 are both OECD-endorsed and described in formal OECD Series reports (Farhat and Kennedy, 2019; Doering et al., 2019). AOP:456, which is currently “under review” on the database, is also accompanied by a peer-reviewed article providing additional context and evidence (Shankar and Villeneuve, 2023). These publications enriched the datasets by providing mechanistic detail, empirical evidence, and contextual information that, in some cases, were not fully captured in the AOP-Wiki entries alone.

An overview of the AOPs represented in the final network, as recorded in the AOP-Wiki at the time of the data collection, is provided in Table 1.

**TABLE 1:** Adverse Outcome Pathways extracted from AOP-Wiki to compose the cardiotoxicity AOP network.

ID	Title	MIE	AO	OECD status	Available at
16	Acetylcholinesterase inhibition leading to acute mortality	Acetylcholinesterase (AchE) inhibition	Increased mortality; decrease, population growth rate	Under development	https://aopwiki.org/aops/16
21	Aryl hydrocarbon receptor activation leading to early life stage mortality, via increased COX-2	Activation, AhR	NA	WPHA/WNT endorsed	https://aopwiki.org/aops/21
94	Sodium channel inhibition leading to congenital malformations	Inhibition, sodium channel	Increased, amputations	NA	https://aopwiki.org/aops/94
138	Organic anion transporter (OAT1) inhibition leading to renal failure and mortality	Inhibition, organic anion transporter 1 (OAT1)	Increased mortality; decrease, population growth rate	NA	https://aopwiki.org/aops/138
104	Altered ion channel activity leading impaired heart function	Impaired, ion channels	Increased mortality	NA	https://aopwiki.org/aops/104
150	Aryl hydrocarbon receptor activation leading to early life stage mortality, via reduced VEGF	Activation, AhR	Increase, early life stage mortality	WPHA/WNT endorsed	https://aopwiki.org/aops/150
177	Cyclooxygenase 1 (COX1) inhibition leading to renal failure and mortality	Inhibition, cyclooxygenase 1 activity	Increased mortality; decrease, population growth rate	NA	https://aopwiki.org/aops/177
261	L-type calcium channel blockade leading to heart failure via decrease in cardiac contractility	Blockade, L-type calcium channels	Heart failure	Under development	https://aopwiki.org/aops/261
433	hERG channel blockade leading to sudden cardiac death	hERG channel blockade	Sudden cardiac death	NA	https://aopwiki.org/aops/433
448	ROS, inflammation, and activation of nAChR lead to increased incidence of cardiovascular morbidity and mortality	NA	NA	NA	https://aopwiki.org/aops/448
456	Aryl hydrocarbon receptor activation leading to early life stage mortality via sox9 repression induced cardiovascular toxicity	Activation, AhR	Increase, early life stage mortality	Under review	https://aopwiki.org/aops/456
479	Mitochondrial complexes inhibition leading to left ventricular function decrease via increased myocardial oxidative stress	Inhibition, mitochondrial electron transport chain complexes	Decrease left ventricular function	Under development	https://aopwiki.org/aops/479
480	Mitochondrial complexes inhibition leading to heart failure via decreased ATP production	Inhibition, mitochondrial electron transport chain complexes	Decrease left ventricular function	Under development	https://aopwiki.org/aops/480

AchE: acetylcholinesterase; AhR: aryl hydrocarbon receptor; AO: adverse outcome; AOP: adverse outcome pathway; COX1: Cyclooxygenase 1; hERG: Human Ether-à-go-go Related Gene; LV: left ventricle; MIE: molecular initiating event; nAChR: nicotinic acetylcholine receptor; OAT1: Organic anion transporter 1; OECD: Organisation for Economic Co-operation and Development; ROS: reactive oxygen species; VEGF: vascular endothelial growth factor; WPHA: working party on hazard assessment; WNT: working group of the national coordinators of the test guidelines programme.

**FIGURE 3:**
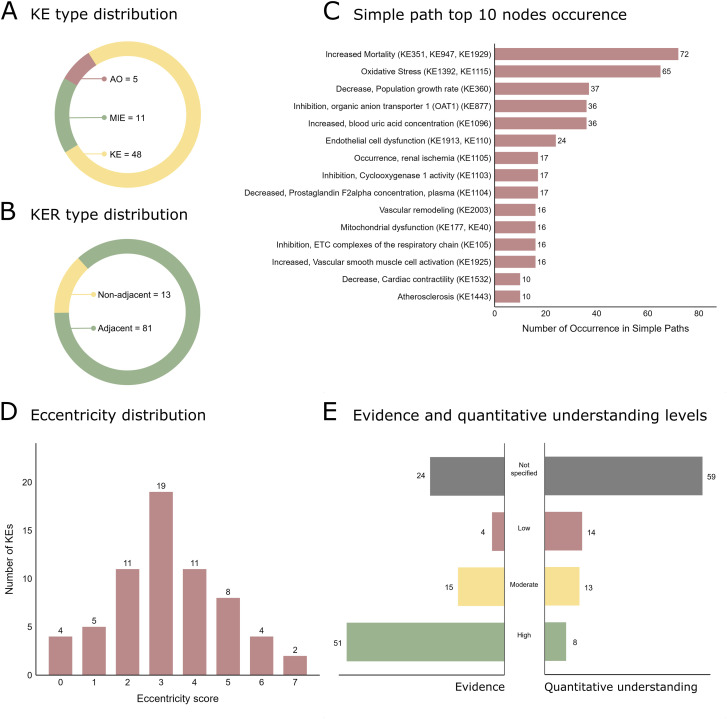
Analysis summary of the cardiotoxicity AOP network. **(A)** Key event type distribution. **(B)** Key event relationship type distribution. **(C)** Top 15 nodes by occurrence in simple paths. **(D)** Eccentricity distribution. **(E)** Evidence and quantitative understanding levels of adjacent and non-adjacent key event relationships. KE: Key events; KER: Key event relationship.

Following KE harmonisation, the final network included 64 nodes and 94 directed edges, representing 11 MIEs, 48 KEs, and 5 AOs (Figure 3A). Adjacent KERs comprised 86.17% (n = 81) of all connections, while 13.83% (n = 13) were non-adjacent links integrated to preserve biological coherence across divergent AOP structures (Figure 3B). KE sharing was predominantly skewed toward single-occurring nodes, yet a subset served as cross-cutting integrators. Of the total 64 KEs, 47 (73.44%) appeared in a single AOP, 10 (15.63%) in two, 5 (7.81%) in three, and one each (1.56%) in four and eight AOPs. “Increased Mortality (KE351, KE947, KE1929)” was the most recurrent (8 AOPs), followed by “Oxidative Stress (KE1392, KE1115)” (4 AOPs). Clusters of KEs sharing three AOPs were observed along the AhR axis (“Activation, AhR”; “Dimerization, AHR/ARNT”) and in cardiovascular function (“Altered, Cardiovascular development/function”; “Decrease, Cardiac contractility”; “Increased, cardiac arrhythmia”) (Supplementary Files).

### 3.2 Structural and topological characterization

Analysis of the network structure demonstrated heterogeneous connectivity patterns with hubs that bridge multiple mechanistic domains. The total degree had a mean of 2.94 and a maximum of 13. The most connected nodes were “Oxidative Stress (KE1392, KE1115)” and “Increased Mortality (KE351, KE947, KE1929)”, both with a total degree of 13 and 12 respectively, followed by extra-cardiac integrators such as “Occurrence, renal proximal tubular necrosis (KE1104)” in 3rd place and “Increased, blood uric acid concentration (KE1096)” in 7th place. These events acted as hubs, bridging multiple mechanistic domains within the cardiotoxicity cascade.

The betweenness centrality ranked “Oxidative Stress (KE1392, KE1115)” as the highest-betweenness node (1.23), followed by “Increased, blood uric acid concentration (KE1096)” (0.49), and “Decrease, Cardiac contractility (KE1532)” (0.39). These nodes lie on many of the shortest paths and are positioned to mediate communication between mechanistic modules. Interestingly, the second-positioned KE and other top-ranked ones in betweenness centrality occur outside the heart, in the kidneys, highlighting the systemic nature of cardiac physiology and the cardio-renal interdependence.

The stress centrality reached its maximum values for key integrative nodes, most notably “Oxidative Stress (KE1392, KE1115)”, “Increased Mortality (KE351, KE947, KE1929)”, and “Endothelial cell dysfunction (KE1913, KE110)”. This pattern indicates that these nodes serve as critical convergence points for a vast number of adverse pathways, consistent with their roles as essential bridges between upstream initiators and downstream outcomes.

To capture multidimensional prominence, we computed a combined importance score. By this index, “Oxidative Stress (KE1392, KE1115)” ranked first, followed by “Increased Mortality (KE351, KE947, KE1929)”, “Decrease, Cardiac contractility (KE1532)”, “Endothelial cell dysfunction (KE1913, KE110)” and “Increased, blood uric acid concentration (KE1096)”. These results indicate that oxidative stress and mortality function simultaneously as mechanistic hubs and critical outcomes within the network.

Complete data on the topographic indicators can be found in Supplementary Data S7.

Directional balance was investigated through convergent/divergent status (Table 2). We identified 14 (21.9%) convergent nodes (downstream recipients with larger inflow), 13 (20.3%) divergent nodes (upstream initiators with larger outflow), and 37 (57.8%) balanced nodes.

**TABLE 2:** List of the identified convergent and divergent key events.

Convergent KEs		Divergent KEs	
Type	Name	Type	Name
AO	Increased mortality	KE	Dimerization, AHR/ARNT
KE	Altered, cardiovascular development/function	KE	Cell injury/death
KE	Occurrence, renal proximal tubular necrosis	KE	Decrease, calcium currents
KE	Atherosclerosis	KE	Increase, slincR expression
KE	Cardiovascular dysregulation	KE	Increased, vascular smooth muscle cell activation
AO	Decrease LV function	KE	Increased, blood uric acid concentration
KE	Decrease, cardiac contractility	KE	Increased, secretion of catecholamine
AO	Decrease, population growth rate	KE	Occurrence, tophi (urate) deposition
KE	Decrease, sox9 expression	KE	Oxidative stress
AO	Heart failure	KE	Endothelial cell dysfunction
KE	Increased, cardiac arrhythmia	KE	Mitochondrial dysfunction
KE	Respiratory distress/arrest	MIE	AchE inhibition
KE	Vascular remodeling	MIE	Activation, AhR
KE	Ventricular remodeling	​	​

AchE: acetylcholinesterase; AhR: aryl hydrocarbon receptor; AO: adverse outcome; KE: key event; LV: left ventricle; MIE: molecular initiating event.

Regarding simple path occurrence, the analysis highlighted a small set of recurrent mediators. As shown in Figure 3C, the fifteen nodes with the highest counts included KEs taking place in the heart as “Oxidative Stress (KE1392, KE1115)”, “Decrease, Cardiac contractility (KE1532)” and “Mitochondrial dysfunction (KE177, KE40)”, as well as renal physiology-related KEs, such as “Increased, blood uric acid concentration (KE1096)” and “Occurrence, renal ischemia (KE1105)”, among others. Top nodes are expected to recur across many mechanistic routes, consistent with their strategic positions along alternative trajectories. These findings suggest moderate network compactness and efficient signal propagation between MIEs and AOs across multiple mechanistic routes.

Eccentricity complements degree- and path-based centralities by quantifying the maximum distance from each key event to its farthest accessible downstream target. This index ranged from 0 to 7, with the majority of nodes clustered between scores of 2 and 4 (Figure 3D). Outcome-level nodes such as “Increased Mortality (KE351, KE947, KE1929)”, “Decrease, Cardiac ejection fraction (KE1533)”, “Increase, Cardiac remodelling (KE2084)” and “Torsades de Pointes (KE 1963)”, among others, showed low eccentricity. This low score confirms their topology as terminal or penultimate sink nodes, representing the endpoints of biological cascades. High-eccentricity nodes, such as “Occurrence, tophi (urate) deposition (KE1102)” and “Inhibition, Cyclooxygenase 1 activity (KE1103)” are peripheral initiators whose effects require multiple steps to reach the final adverse outcomes.

### 3.3 Key event relationships: evidence and quantitative understanding

Assessment of KER evidence and quantitative understanding included adjacent and non-adjacent relationships for a broader evaluation of the network. Evidence levels revealed that 54.26% (n = 51) were supported by high or strong evidence, 15.96% (n = 15) had moderate support, 4.25% (n = 4) presented low levels of evidence, while 25.53% (n = 24) lacked specified evidence levels (Figure 3E). Similarly, the degree of quantitative understanding was limited, with only 37.2% (n = 35) of KERs associated with any quantitative level of understanding. This highlights persistent gaps that may hinder mechanistic modelling and prediction efforts. Full distribution of these data is illustrated in Figure 3E.

**FIGURE 4:**
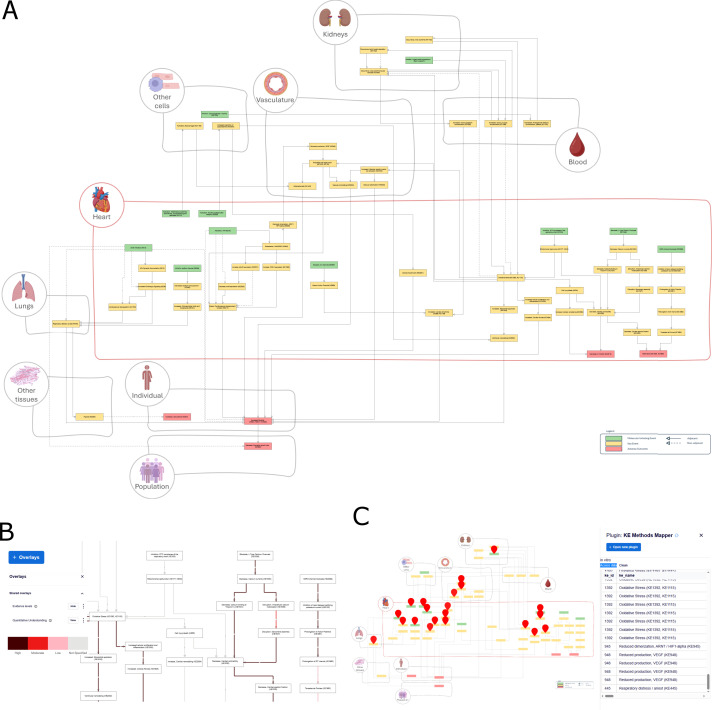
Overview of the interactive cardiotoxicity AOP network model on the MINERVA platform.” **(A)** Overview of the platform displaying the network. **(B)** Example of data overlay visualisation, highlighting different levels of evidence support to the key event relationships. **(C)** KE Methods Mapper plugin overview, showing how the methods catalogue is mapped on the AOP network. The search bar allows filtering the visualized mapped information by key event (KE) name, ID or type, detection method, and key characteristics of cardiovascular toxicants (KE stressors). The platform allows users to access the dedicated AOP-Wiki pages via hyperlinks. Created in BioRender. Ivanets, M. (https://BioRender.com/jpbbquj) is licensed under CC BY 4.0.

These data were prepared as overlays for visualisation on the interactive network displayed in MINERVA, and can be accessed via the “Overlays” feature at https://cardiotox.elixir-luxembourg.org/. Figure 4B displays an example of how KER data - evidence levels in the figure - is visualised at the platform.

### 3.4 Biological key characteristics of network stressors

Fifty KEs (78.13%) were related to key characteristics of cardiovascular toxicants, including KEs, MIEs and one AO. There were 14 (21.87%) KEs not matched, comprising 4 AOs (6.25%), 2 cardiac events (3.12%) and 8 events related to non-cardiac tissues (12.5%). The mapping is described in Supplementary Table S1.

### 3.5 Cataloguing KE measurement assays

By analysing the 71 unique KE ID entries (after harmonisation, the total number of KEs was 64) on AOP-Wiki, we gathered the methods used to monitor or quantify the endpoints. Out of the 64 unique KEs, 43.75% (n = 28) had detection methods explicitly described in the AOP-Wiki. These included *in vivo*, *in vitro* and *in silico* methodologies, such as biochemical assays, imaging, electrophysiological recordings, and molecular biomarkers (Supplementary Data S9). The first version of this catalogue is fully based on what AOP authors reported in the KE entries and does not represent all the potential methods to quantify a KE. However, as a living resource, it is subject to subsequent updates that will help fill the methodological gaps in itself and potentially in AOP-Wiki. At this stage, it serves as a starting point for developing an integrated test battery for cardiotoxicity assessment.

A MINERVA platform plugin (Figure 4C) was developed for dynamic visualisation and search of methods for the KEs present in this network. It maps the catalogue on top of the network and facilitates on-demand method mapping. The plugin and its source code can be found at https://github.com/luiz-ladeira/cardiotox_aop_minerva_plugin. To use it, the user can load the plugin directly on the MINERVA platform as described in the methods section.

### 3.6 Interactive visualization and contextual annotations

The complete AOP network was made accessible through a MINERVA-based interactive platform (https://cardiotox.elixir-luxembourg.org/). The two self-loops (“Oxidative Stress” and “Mitochondrial dysfunction”) present in the network are not represented in the interactive version due to CellDesigner software limitations. Nodes were spatially organised according to biological scale (cellular, tissue, organ) related to their contextual metadata (Supplementary Data S6). To improve clarity, an adaptation of the Systems Biology Graphical Notation (SBGN) Activity Flow (AF) language (Mi et al., 2015; Novère et al., 2009) was implemented, in which the rectangle shape represented a KE (equivalent to Biological Activity in SBGN-AF), a directed solid arrow was used for adjacent KERs, and a directed dotted arrow was used for non-adjacent KERs, indicating the flow of information. Figure 4A shows an overview of the network as displayed in the MINERVA platform.

## 4 Discussion and conclusions

The AOP framework is a valuable tool for organising mechanistic knowledge to support chemical safety assessment, particularly through the development of non-animal new approach methodologies (NAMs) (Jaylet et al., 2024; OECD, 2017). In this study, we present and analyse the first AOP-Wiki-derived network for cardiotoxicity, providing a structured overview of a diverse range of mechanisms leading to cardiac damage. The current network is not intended to be the most comprehensive collection of mechanistic understanding on cardiotoxicity mode-of-action, as it is built from linear AOPs available in AOP-Wiki. This resource is intended as a living document, capable of being iteratively updated as new AOPs linking chemical exposure to cardiac outcomes are identified. Incorporating evidence from optimised review methodologies (Van Ertvelde et al., 2023) and data-driven approaches for KE and KER discovery (Gadaleta et al., 2024; Ortega-Vallbona et al., 2024) will progressively expand its scope and improve mechanistic understanding of how chemicals affect the heart.

Nevertheless, our analysis of the AOP-Wiki-derived network revealed an emerging landscape for cardiotoxicity in the database. Most of the KEs identified (n = 47; 73.4%) are part of only one AOP, indicating limited interconnectivity within the current set of cardiotoxicity-related pathways. Yet, the topology still exposed a small set of cross-cutting KEs that organise information flow across modules. For example, “Oxidative Stress (KE1392, KE1115)” consistently ranked at the top or among the highest in degree, betweenness, stress, and simple-path occurrence, and also led the combined importance score. Oxidative stress is a complex imbalance between reactive oxygen species (ROS) production and antioxidant capacity; while transient ROS is involved in redox signalling, prolonged elevation drives disease progression (Carrasco et al., 2021). Mechanistically, this aligns with the well-established role of ROS imbalance as a determinant of cardiac injury (Carrasco et al., 2021). This finding is also consistent with the central role of oxidative stress in cardiotoxicity induced by anthracyclines like doxorubicin, idarubicin, and daunorubicin (Mamoshina et al., 2021) and environmental toxicants such as heavy metals (e.g., lead, cadmium, arsenic) (Ozarde et al., 2025; Pan et al., 2024). From heavy metals, cardiovascular risk is induced via excessive ROS generation, overwhelming natural antioxidant defences, leading to endothelial dysfunction, vascular inflammation, and ultimately, hypertension and atherosclerosis (Ozarde et al., 2025; Pan et al., 2024). Differently, anthracyclines trigger mitochondrial dysfunctions leading to hypoxia and myocardial fibrosis, ultimately developing into heart failure (Carrasco et al., 2021). Similarly, oxidative stress, along with mitochondrial dysfunction, was identified as one of the most highly connected and central key events in AOP networks for hepatotoxicity (Van Ertvelde et al., 2023; Verhoeven et al., 2024), neurotoxicity (Spinu et al., 2019) and nephrotoxicity (Barnes et al., 2024), showcasing it as a fundamental pillar of cellular toxicity across different organs.

Within the heart-related KEs in our network, “Decrease, Cardiac contractility (KE1532)” and “Mitochondrial dysfunction (KE177, KE40)”, joined oxidative stress as a functional triad of hubs. All three had high total degree and betweenness and were repeatedly visited along MIE to AO simple paths. Their co-occurrence is mechanistically coherent: mitochondrial impairment increases ROS and compromises ATP supply; both processes depress excitation-contraction coupling and sarcomeric function, reducing contractile output (Carrasco et al., 2021; Varga et al., 2015). These KEs are therefore sensitive integrators of injury signals from diverse initiators (e.g., ion-channel perturbations, AhR activation, electron transport chain inhibition). At the same time, they lack mode-of-action specificity, an important consideration for interpreting positive signals in screening and for deciding which complementary, more specific readouts to pair with them.

In this sense, the Key Characteristics (KCs) concept helps in contextualising the usually “generic cytotoxic KEs” into organ-specific scenarios and provides critical context for interpreting the topological importance of specific events within the cardiotoxicity network. While AOPs and their related KEs are developed to be “chemical-agnostic” (Vinken et al., 2017), which means any stressor that could trigger a specific MIE within an AOP can be considered an initiator, the KCs concept is rooted in the biological properties that chemicals exhibit (Lind et al., 2021). As one relates to the causality in the biology of perturbation (AOPs) and the other to individual biological mechanisms that chemicals can trigger without sequential causality across biological levels (KCs), KEs can relate to the KCs by their definitions. This is expected to facilitate organ-specific hazard identification and improve the systematic evaluation of mechanistic data for cardiac toxicants. From our network, cross-mapping KEs with KCs of cardiovascular toxicants (even though it was not our primary goal to investigate the vascular aspects of cardiotoxicity) enhances the context-specific interpretation, especially when analysing general cytotoxic events like oxidative stress, mitochondrial dysfunction or cell injury/death and apoptosis, which most of the time are shared mechanisms across different organ toxicities, as found when comparing our network analysis results with other AOP mapping efforts (not only from AOP-Wiki) (Arnesdotter et al., 2021; Barnes et al., 2024; Schaffert et al., 2025; Spinu et al., 2019; Van Ertvelde et al., 2023; Verhoeven et al., 2024; Wiklund et al., 2023). Indeed, from a network topology perspective, the high degree and betweenness centrality of KEs such as oxidative stress and mitochondrial dysfunction, which correspond to KC10 (Induces oxidative stress) and KC8 (Impairs mitochondrial function), do not diminish their utility for organ-specific assessment (Villeneuve et al., 2018). Rather, these highly connected KEs represent critical integrative biological signals through which multiple upstream pathways must pass to reach adverse outcomes. Consequently, while these shared mechanisms converge on common key events across tissues, their organ-specific manifestation is dictated by context-sensitive variations in molecular pathway wiring and quantitative dose-response relationships (Sturla et al., 2014). This means that the probability of exceeding a critical biological tipping point, from adaptive response to pathological injury, is governed by tissue-specific protein isoforms, functional properties, and tissue-specific regulatory network architectures. Altogether, these determine the exposure thresholds at which general stress responses transition to organ-specific adverse outcomes (Maertens et al., 2025).

Outcome-related nodes, including “Increased Mortality (KE351, KE947, KE 1929)”, “Decrease, Cardiac ejection fraction (KE1533)”, “Increase, Cardiac remodelling (KE 2084)”, “Hypoxia (KE590)” and “Torsades de Pointes (KE1963)”, displayed low eccentricity, indicating they are or they are close to terminal endpoints. Among these, “Increased Mortality” further exhibited high stress centrality, confirming its central role in the network where most upstream routes converge. In contrast, several initiator-proximal or extra-cardiac KEs (e.g., “Inhibition, Cyclooxygenase 1 activity (KE1103)”; “Occurrence, tophi (urate) deposition (KE1102)”) sat at the network periphery with higher eccentricity, consistent with their role as upstream or system-level contributors rather than heart-intrinsic drivers. That is, these nodes are more distant from others in the network, reflecting their role as early or external triggers rather than direct cardiac effectors.

The KC framework complements the AOP network interpretation by also illuminating why certain KEs with lower statistical-based topological scores remain essential for mechanistic interpretation. Cardiac-specific KEs such as hERG channel blockade (KE 2099), Decrease, Calcium currents (KE1530), and Torsades de Pointes (KE 1963), which mapped to KC1 (Impairs regulation of cardiac excitability), exhibit lower betweenness centrality but provide diagnostic resolution that general stress markers cannot offer. While a positive signal in general oxidative stress assays confirms the presence of cellular redox imbalance, these endpoints often lack the mechanistic resolution to differentiate between direct pro-oxidants and compounds that elicit ROS secondary to mitochondrial dysfunction or calcium overload (Mamoshina et al., 2021; Varga et al., 2015). In contrast, detecting hERG blockade or decreased calcium currents directly identifies specific proarrhythmic liabilities (Lester, 2021). Thus, an effective testing battery must pair highly connected, non-specific KEs (broad coverage and high sensitivity) with cardiac-specific, mechanistically diagnostic KEs (high specificity) (Clark et al., 2022).

A notable and recurrent feature was the systemic integration of the heart, especially the cardio-renal axis, illustrated in our interactive and contextual version of the network. Extra-cardiac KEs such as “Occurrence, renal proximal tubular necrosis (KE1097)” and “Increased, blood uric acid concentration (KE1096)” were among the most connected and ranked near the top in betweenness and total degree, indicating high connectivity. These patterns support a mechanistic bridge whereby renal injury and urate dysregulation amplify systemic oxidative and inflammatory burden that exacerbates cardiac dysfunction (Ding et al., 2022; Ozarde et al., 2025). In practice, this means a subset of cardiotoxic trajectories are genuinely systemic and may be missed by single-organ assays. The cardio-renal axis exemplifies a bidirectional scenario in which dysfunction in the kidney or heart triggers pathophysiological feedback loops that accelerate damage in both organs. This cross-talk is mediated by haemodynamic shifts, neurohormonal activation (particularly of the renin-angiotensin-aldosterone system and sympathetic overactivity), and circulating inflammatory and fibrotic factors, including protein-bound uremic toxins such as indoxyl sulphate, which exert direct pro-hypertrophic and pro-fibrotic effects on cardiac tissue (Lekawanvijit et al., 2012; Rangaswami et al., 2019). The network therefore provides a rationale for multi-organ microphysiological systems (MPS) or staged testing strategies when cardio-renal axes are implicated (Daley et al., 2022; OECD, 2017). On the other hand, the only KC not represented in our network was KC7 (Causes dyslipidaemia), which is related to LDL secretion in the liver and nutrient absorption in the gut, highlighting not only a gap in the current network, mostly due to scoping criteria, but also an opportunity while designing comprehensive MPS and indicating potential curation priorities to AOP developers.

Edge-type composition also carries insight. While the majority of KERs were adjacent (86.17%), a meaningful subset were non-adjacent (13.83%). These long-range links often connect early molecular events to organ-level dysfunction and likely compress intermediate biology that is either context-dependent or not yet curated in AOP-Wiki. They are useful for scoping hazards and tracing plausible routes to outcomes, but they simultaneously flag the most impactful knowledge gaps for future curation and quantification (Villeneuve et al., 2018). The evidence overlays corroborate this: just over half of KERs carry high/strong evidence, but fewer than 40% include any quantitative understanding. Notably, quantitative links are sparse precisely around the hub KEs, where they would most enable quantitative AOP (qAOP) development, sensitivity analyses, and extrapolation (OECD, 2017; Spinu et al., 2020).

Together, these structural and evidential features converge on a clear message. First, oxidative stress, mitochondrial dysfunction, and decreased contractility form a core, recurrent axis across mechanistic routes from diverse initiators to cardiac adverse outcomes; they should be prioritised for measurement and, where possible, for quantitative linkage. Second, cardio-renal crosstalk is not incidental: kidney-centred KEs frequently mediate routes to cardiac outcomes and warrant explicit consideration when designing evaluation strategies. Third, while hub KEs are powerful integrators, they require pairing with more specific, pathway-diagnostic readouts to improve mode-of-action resolution. These conclusions directly inform the design principles of a mechanistically anchored, animal-free cardiotoxicity testing battery, as discussed next.

### 4.1 From AOP network to an animal-free cardiotoxicity testing battery

A primary goal of developing this network is to inform the design of a mechanistically anchored, animal-free test battery for cardiotoxicity prediction. To this end, we systematically reviewed the methods section of the KE pages at AOP-Wiki and extracted the reported methods to quantify and monitor individual KEs. Explicit detection methods were listed for only 43.75% of unique KEs, pointing to a scattered knowledge base. The documented approaches spanned *in vivo*, *in vitro*, and *in silico* methods, including biochemical assays, imaging, and electrophysiological readouts. While this catalogue provides a foundational starting point, it is not exhaustive and reflects the current state of user-based reporting within the AOP-Wiki, which itself has limitations. A recent analysis of the AOP-Wiki showed that fields describing supporting evidence are often incomplete (Jaylet et al., 2024), which likely extends to the methodologies for measuring KEs. It is worth noting that Lind et al. (2021) also lists detection methods and stressors leading to KCs, which complements our methods catalogue.

Despite these gaps, the AOP network and the related KCs helps prioritise what to measure. Hub KEs shared across multiple AOPs are efficient assay targets, but specificity is also essential for an informative battery. Non-specific KEs, such as oxidative stress, mitochondrial dysfunction, and cytotoxicity, are common across organ systems and chemical classes and cannot, on their own, discriminate specific cardiotoxic liability (Arnesdotter et al., 2021; Barnes et al., 2024; Mamoshina et al., 2021). In contrast, cardiac-specific KEs provide higher diagnostic value: hERG/IKr blockade is the canonical driver of drug-induced QT prolongation and torsades de pointes risk, while additional mechanisms include inhibition of cardiomyocyte survival signalling (e.g., VEGF/EGF pathways) and perturbations of contractility or calcium handling (Clark et al., 2022; Lester, 2021; Mamoshina et al., 2021). Accordingly, an effective test battery should follow an Integrated Approaches to Testing and Assessment (IATA) logic, combining general cellular-health assays (e.g., viability, mitochondrial function) with targeted tests of cardiac-specific mechanisms (e.g., multi-ion channel panels, contractility, calcium cycling), to cover the relevant mechanism space (Daley et al., 2022; OECD, 2017; 2020; Schaffert et al., 2023).

In this sense, the ALTERNATIVE project has developed an AOP-informed IATA that integrates data from human-relevant *in vitro* assays, transcriptomics, and high-content screening to evaluate environmental chemicals’ toxicity (Schaffert, Murugadoss, et al., 2024; Schaffert, Paparella, et al., 2024). This framework supports evaluating cardiotoxic liability through both general and cardiac-specific endpoints, structured along mechanistic axes such as contractility, electrophysiology, and cell viability. Nevertheless, unmet opportunities for including other molecular strategies could still be explored, especially to detect mixture or multi-target interaction outcomes, quantitative links from KEs to phenotypic dynamics, and explicit sources of biological variability in predictions. Considering a broader scenario of mechanism possibilities, by incorporating nuclear and membrane receptors and compound metabolism pathways, among other modulators, would further enrich the current understanding and help uncover novel modes of action. This holistic inclusion could capture compound- and population-specific vulnerabilities, thereby advancing the refinement of predictive, human-relevant cardiotoxicity testing.

Mechanistic compound controls (i.e., positive compound controls with well-described mechanisms of action) are essential to calibrate and validate such a battery (Verhoeven et al., 2025). Compounds with well-characterised modes of action allow sensitivity/specificity checks and enable cross-platform comparisons (Parish et al., 2020). For instance, moxifloxacin serves as a standard positive control for reproducible and multi-method comparisons of QT intervals (Darpo et al., 2006); doxorubicin anchors structural cardiotoxicity via mitochondrial damage and oxidative stress (Chen et al., 2023); and verapamil illustrates that hERG block does not necessarily translate into proarrhythmia due to compensatory calcium-current effects (Daley et al., 2022). Curated drug sets, as from the Comprehensive *in vitro* Proarrhythmia Assay (CiPA), further link assay outputs to established clinical outcomes and strengthen external validity (Konala et al., 2025). Similarly, the United States Food and Drug Administration (FDA)-labelling-based Drug-Induced Cardiotoxicity rank (DICTrank) compendium (1,318 drugs; severity linked to the Boxed Warning, Warnings and Precautions, and Adverse Reactions sections of FDA labels) offers a clinically grounded reference that shows high concordance with the Drug-Induced QT prolongation risk Assessment (DIQTA) database and the CredibleMeds clinical risk classification (Qu et al., 2023). The cardiotoxicity AOP network provides a mechanistic scaffold to interpret these control compounds in a broader biological context. It enables the mapping of compound-induced events to specific key events and pathways, clarifying the coverage of relevant mechanisms across assays. This not only supports rational selection and interpretation of positive controls but also the comparison and benchmarking of new test systems to clinically established mechanisms.

Alignment with regulatory expectations is also required. In the pharmaceutical domain, cardiotoxicity assessment is underpinned by the International Council for Harmonisation of Technical Requirements for Pharmaceuticals for Human Use - ICH safety pharmacology and proarrhythmia guidelines (FDA, 2005; FDA, 2021). ICH S7A defines the core safety-pharmacology battery and general principles, including dedicated cardiovascular evaluations to protect trial participants while minimising animal use (FDA, 2021). ICH S7B specifies a nonclinical strategy centred on *in vitro* IKr (hERG) assays and *in vivo* QT studies, supported by an integrated risk assessment to judge delayed ventricular repolarisation risk (FDA, 2005). Clinically, ICH E14 established the thorough QT paradigm and formalised links between nonclinical findings and clinical proarrhythmic risk (EMA, 2022). Together, these guidances constitute a mature, QT-centric framework that has reduced proarrhythmic liabilities; however, it can be conservative, and recent updates recognising “double-negative” outcomes in best-practice nonclinical assays signal a gradual shift toward integrated, mechanism-based assessments (Lester, 2021). By contrast, current guidelines for industrial chemicals, pesticides and biocides largely neglect functional cardiotoxicity, focusing instead on endpoints such as heart weight and histopathology from animal studies (Schaffert et al., 2023). OECD has advanced IATA and the use of AOPs to organise non-animal evidence for decision-making, including guidance on IATA concepts, reporting of defined approaches and case-study programmes. Acceptance and validation principles for new methods are codified in an OECD Guidance Document, supporting international uptake once fitness-for-purpose is demonstrated (OECD, 2017). Nonetheless, the lack of quantitative key-event relationship data remains a barrier to regulatory utility, underscoring the need for quantitative integration if functional cardiotoxicity is to be addressed systematically and mechanistically.

NAMs based on human-induced pluripotent stem cell-derived cardiomyocytes (hiPSC-CMs) and cardiac organoids enable high-throughput, human-relevant assessment of cardiac liabilities (Daley et al., 2022; Gisone et al., 2022). Within our network, three hub KEs emerged in the heart network: oxidative stress, mitochondrial dysfunction and decreased contractility, and each can be measured with established assays. For decreased contractility, hiPSC-CMs provide functional readouts such as beating frequency, contraction amplitude and calcium handling, capturing disturbances in both electrophysiological and contractile function (Dou et al., 2022). Mitochondrial dysfunction can be detected via membrane potential (e.g., using tetramethylrhodamine ethyl ester perchlorate - TMRE fluorescent probe), oxygen consumption rate (e.g., Seahorse extracellular flux analysis) and ATP production (e.g., luciferase-based assays), endpoints particularly relevant for energetics-impairing compounds (e.g., anthracyclines) (Sharma et al., 2025). Oxidative stress can be quantified by ROS formation, lipid peroxidation and alterations in antioxidant enzyme dynamics (Murphy et al., 2022). Within *in vitro* setups, hiPSC-CMs and cardiac organoids also provide a rich platform for omics data collection (Verheijen et al., 2022) and AOPs can provide the mechanistic scaffold for data interpretation. The feasibility of such approach has been increasingly demonstrated by different studies, including “molecular AOPs”, which uses transcriptomics data to provide quantitative biological context to KEs, leveraging AOP-Wiki ontology annotations to KEs (Martens et al., 2023). In this direction, integrating omics data facilitates the transition of static AOP networks to dynamic quantitative AOPs (qAOP). Recent advancements in benchmark-dose modelling and AOP enrichment enable the derivation of transcriptomic-based points of departure, proving mechanistically grounded explanations for chemical potency and hazard prioritisation (Serra et al., 2025; Vuong et al., 2025).

Combining these complementary readouts enables a battery that captures convergent mechanisms of cardiotoxicity. This strategy moves beyond single-endpoint assays such as hERG, which although sensitive, lack specificity and mechanistic breadth (Daley et al., 2022). CiPA already demonstrated the value of integrating multiple ion-channel assays with *in silico* modelling (Lester, 2021). Our AOP network supports extending this principle to non-electrophysiological endpoints essential for detecting structural, contractile and metabolic toxicities and a scaffold for different omics integration approaches in the cardiac context. In sum, our AOP network provides the organising principle for designing an animal-free test battery and the curated catalogue of KE measurement methods identifies immediate assay candidates and evidence gaps.

### 4.2 Challenges, opportunities and future perspectives

A significant challenge encountered during this work was the lack of standardisation in the AOP-Wiki, a scenario also identified in other mapping studies (Jaylet et al., 2024). Many KEs describing the same biological process were titled differently, requiring manual harmonisation to build a coherent network. For instance, events with overlapping biological scope were recorded under multiple names and with different levels of detail, which fragments knowledge and complicates automated network generation. These redundancies have been explicitly noted for ROS-related KEs, where multiple nuanced entries exist despite referring to similar processes, prompting the launch of the “Mystery of ROS” initiative to create harmonised consensus KEs (Tanabe et al., 2022; Tanabe et al., 2023). Such efforts highlight the broader need for ontology-based alignment of KE terminology, ensuring consistency in descriptors like directionality, biological objects, and processes to make entries machine-readable and interoperable. This demand has been echoed by the Society for the Advancement of AOPs Knowledgebase Interest Group (SKIG), which has prioritised ontology-driven harmonisation, systematic curation, and development of umbrella KEs as central steps towards AOP-Wiki 3.0 (Wittwehr et al., 2025). In parallel, the recently launched SCAHT AOP_HUB provides a practical forum for training, knowledge exchange, and discussion of recurring challenges in AOP development, with a strong focus on engaging early-career researchers (Coerek et al., 2025). Together, these initiatives demonstrate that the community is actively addressing the standardisation bottleneck and moving towards a more interoperable and predictive AOP knowledge base.

Furthermore, the current AOP framework in the AOP-Wiki insufficiently captures feedback loops and compensatory mechanisms, which are essential to understand the dose- and time-dependent nature of cardiotoxicity (Georgiadis et al., 2022; Mamoshina et al., 2021). Linear AOPs, by design, tend to oversimplify complex biological responses, whereas AOP networks offer a more realistic systems-level representation of toxicity by accounting for converging and diverging pathways (Sewell et al., 2018). Our network begins to address this by linking multiple MIEs to shared KEs, but a more explicit integration of dynamic motifs - such as feedback and feedforward regulation, as highlighted in AOP network development frameworks (Knapen et al., 2018; Villeneuve et al., 2018) - would significantly enhance its predictive power. In this direction, the Disease Maps community (https://disease-maps.io/) has demonstrated that mechanistic, highly detailed molecular maps enable systematic integration of multi-omics data and contextual interpretation of dynamic processes, providing a promising blueprint for advancing AOPs beyond static representations and tools for benchmarking existing networks (Ladeira et al., 2025; Mazein et al., 2018; Staumont et al., 2025). Moving from the mostly high-level representation of biological activities in the AOP-Wiki towards more detailed molecular maps would improve interpretability of experimental outcomes and mechanistic grounding. This does not require enforcing excessive pathway granularity but can be achieved through complementary strategies such as ontology mapping, pathway enrichment analysis, and the application of computational approaches for quantitative AOP modelling (Jaylet et al., 2024; Knapen et al., 2018).

Importantly, such advances depend on making AOP knowledge not only interoperable but also machine-actionable. Recent work has stressed that adherence to the FAIR principles (ensuring that AOPs are Findable, Accessible, Interoperable, and Reusable - FAIR) is essential to increase their visibility, credibility, and uptake in regulatory decision-making (Wittwehr et al., 2023). FAIRification enables automation, provenance tracking, and semantic alignment, addressing current issues of network fragmentation and enhancing trust in AOP-derived models. In this context, detailed molecular mapping does not merely increase complexity but creates opportunities for advanced visualisation, data integration, and predictive modelling. A recent example is the explorable model of cytokine release syndrome developed by Mazein et al. (2025), which integrates multiple AOPs induced by immunomodulatory therapies into a molecular-level systems biology map. By combining diagrammatic KE representations with machine-readable formats (e.g., SBML, SBGN, stable identifiers), this resource ensures reproducibility and interoperability while enabling hypothesis generation, biomarker discovery, and mechanistic modelling. Collectively, these directions underscore the need for cross-disciplinary integration of systems biology and toxicology to make AOP-based models more dynamic, predictive, FAIR and regulatory-relevant.

A major opportunity ahead is the transition from qualitative AOP networks to qAOP models. By parameterising key event relationships with dose-response and time-course data, qAOPs can predict the magnitude or likelihood of an adverse outcome from defined perturbations at the molecular level (Conolly et al., 2017; Spinu et al., 2020). By quantifying KERs and network-level parameters, qAOPs also provide the quantitative context that helps in interpreting general cytotoxic events in different organs and systems. Such dynamic models can be implemented through diverse approaches, from Bayesian and regression-based methods to mechanistic ODE frameworks or logic-based modelling, chosen according to data availability and context-of-use (Paini et al., 2022; Perkins et al., 2019). When coupled with physiologically based pharmacokinetic or quantitative systems pharmacology models, they enable quantitative in vitro-to-in vivo extrapolation, translating cell-based assay data into exposure thresholds relevant for humans (Proença et al., 2021; Sang et al., 2024). This integrative direction has already demonstrated predictive capacity for drug-induced cardiotoxicity (Sang et al., 2024), underscoring its potential for translational applications.

### 4.3 Concluding remarks

This study provides the first integrated AOP network for cardiotoxicity, in addition to mapping the KEs to key characteristics of cardiovascular toxicants, transforming the current knowledge in the OECD AOP-Wiki into a coherent map of cardiac hazard. The analysis identified oxidative stress, mitochondrial dysfunction, and decreased contractility as central, highly connected key events that serve as the primary convergence points of diverse chemical stressors. Importantly, the network highlights the systemic nature of cardiotoxicity, with the cardio-renal axis acting as a mechanistic bridge that is not captured by channel-centric assays, while identifying mechanistic gaps relevant to capture the broad chemical space of cardiotoxic chemicals. These findings provide a strong mechanistic foundation for designing an animal-free and human-relevant test battery using a diverse collection of methods, including multi-organ models to address complex mechanisms. While the sparsity in the source data highlights the need for greater standardisation and curation within the AOP community, our work demonstrates the power of the AOP network framework to synthesise complex toxicological information and serves as a guiding resource for IATA design and the prioritisation of animal-free testing strategies. Our KE-assay catalogue and the FAIR-aligned interactive and contextual map provide the community with a living scaffold that can be iteratively refined with emerging evidence. Future work should evolve the networks into qAOPs integrated with detailed systems-biology maps to create dynamic tools for assessing cardiovascular hazard and risk and ensuring the safety of both new drugs and environmental chemicals.

## Data Availability

The datasets are available on GitHub (https://github.com/luiz-ladeira/cardiotox_aop_minerva_plugin) and on the MINERVA platform (https://cardiotox.elixir-luxembourg.org/), under the Creative Commons Attribution 4.0 International (CC BY 4.0) License (https://creativecommons.org/licenses/by/4.0/).

## Glossary

AchE: Acetylcholinesterase
AF: Activity Flow
AhR: Aryl hydrocarbon receptor
AO: Adverse Outcome
AOP: Adverse Outcome Pathway
CiPA: Comprehensive *in vitro* Proarrhythmia Assay
COX-2: Cyclooxygenase-2
COX1: Cyclooxygenase 1
DICTrank: Drug-Induced Cardiotoxicity rank
DIQTA: Drug-Induced QT prolongation risk Assessment
EGF: Epidermal Growth Factor
FAIR: Findable, Accessible, Interoperable, and Reusable
FDA: Food and Drug Administration
hERG: Human Ether-à-go-go Related Gene
hiPSC-CMs: Human-induced pluripotent stem cell-derived cardiomyocytes
IATA: Integrated Approaches to Testing and Assessment
ICH: International Council for Harmonisation of Technical Requirements for Pharmaceuticals for Human Use
IKr: Rapid component of the delayed rectifier potassium current
KC: Key Characteristics
KE: Key Event
KERs: Key Event Relationships
LV: Left Ventricle
MIE: Molecular Initiating Event
MINERVA: Molecular Interaction NEtwoRk VisuAlization (platform)
MPS: Microphysiological systems
nAChR: Nicotinic Acetylcholine Receptor
NAMs: New Approach Methodologies
OAT1: Organic anion transporter 1
OECD: Organisation for Economic Co-operation and Development
qAOP: Quantitative Adverse Outcome Pathway
QT/QTc: QT interval/corrected QT interval
ROS: Reactive Oxygen Species
SBGN: Systems Biology Graphical Notation
SBML: Systems Biology Markup Language
SKIG: Society for the Advancement of AOPs Knowledgebase Interest Group
TMRE: Tetramethylrhodamine ethyl ester perchlorate
VEGF: Vascular Endothelial Growth Factor
WPHA: Working Party on Hazard Assessment
WNT: Working Group of the National Coordinators of the Test Guidelines Programme

## References

[B1] Ankley G. T. Bennett R. S. Erickson R. J. Hoff D. J. Hornung M. W. Johnson R. D. (2009). Adverse outcome pathways: a conceptual framework to support ecotoxicology research and risk assessment. Environ. Toxicol. Chem. 29 (3), 730–741. 10.1002/etc.34 20821501

[B2] Arnesdotter E. Spinu N. Firman J. Ebbrell D. Cronin M. T. D. Vanhaecke T. (2021). Derivation, characterisation and analysis of an adverse outcome pathway network for human hepatotoxicity. Toxicology 459, 152856. 10.1016/j.tox.2021.152856 34252478

[B3] Bajard L. Adamovsky O. Audouze K. Baken K. Barouki R. Beltman J. B. (2023). Application of AOPs to assist regulatory assessment of chemical risks – case studies, needs and recommendations. Environ. Res. 217, 114650. 10.1016/j.envres.2022.114650 36309218 PMC9850416

[B4] Barnes D. A. Firman J. W. Belfield S. J. Cronin M. T. D. Vinken M. Janssen M. J. (2024). Development of an adverse outcome pathway network for nephrotoxicity. Archives Toxicol. 98 (3), 929–942. 10.1007/s00204-023-03637-7 PMC10861692 38197913

[B5] Carrasco R. Castillo R. L. Gormaz J. G. Carrillo M. Thavendiranathan P. (2021). Role of oxidative stress in the mechanisms of anthracycline‐induced cardiotoxicity: effects of preventive strategies. Oxidative Med. Cell. Longev. 2021 (1), 8863789. 10.1155/2021/8863789 33574985 PMC7857913

[B6] Chen R. Niu M. Hu X. He Y. (2023). Targeting mitochondrial dynamics proteins for the treatment of doxorubicin-induced cardiotoxicity. Front. Mol. Biosci. 10, 1241225. 10.3389/fmolb.2023.1241225 37602332 PMC10437218

[B7] Clark A. P. Wei S. Kalola D. Krogh‐Madsen T. Christini D. J. (2022). An in Silico–In vitro pipeline for drug cardiotoxicity screening identifies ionic pro‐arrhythmia mechanisms. Br. J. Pharmacol. 179 (20), 4829–4843. 10.1111/bph.15915 35781252 PMC9489646

[B8] Coerek E. Kuchovska E. Gerner L. Nilma L. Villeneuve D. Fritsche E. (2025). The SCAHT adverse outcome pathway (AOP)_HUB: a hands-on platform for information exchange, sharing, and developing AOPs. ALTEX 42 (2), 361–362. 10.14573/altex.2502051 40235188

[B9] Conolly R. B. Ankley G. T. Cheng W. Mayo M. L. Miller D. H. Perkins E. J. (2017). Quantitative adverse outcome pathways and their application to predictive toxicology. Environ. Sci. and Technol. 51 (8), 4661–4672. 10.1021/acs.est.6b06230 28355063 PMC6134852

[B10] Daley M. Mende U. Choi B.-R. McMullen P. D. Coulombe K. L. K. (2022). Beyond pharmaceuticals: fit-For-Purpose new approach methodologies for environmental cardiotoxicity testing. ALTEX 40, 103–116. 10.14573/altex.2109131 35648122 PMC10502740

[B11] Darpo B. Nebout T. Sager P. T. (2006). Clinical evaluation of QT/QTc prolongation and proarrhythmic potential for nonantiarrhythmic drugs: the international conference on harmonization of technical requirements for registration of pharmaceuticals for human use E14 guideline. J. Clin. Pharmacol. 46 (5), 498–507. 10.1177/0091270006286436 16638733

[B12] Ding R. Ren X. Sun Q. Sun Z. Duan J. (2022). An integral perspective of canonical cigarette and e-cigarette-related cardiovascular toxicity based on the adverse outcome pathway framework. J. Adv. Res. 48, S209012322200193X. 10.1016/j.jare.2022.08.012 35998874 PMC10248804

[B13] Doering J. Hecker M. Zhang X. (2019). Adverse outcome pathway on aryl hydrocarbon receptor activation leading to early life stage mortality, via increased COX-2 OECD series on adverse outcome pathways no. 12; OECD series on adverse outcome pathways. Paris: OECD Publishing. 10.1787/bd46b538-en

[B14] Dou W. Malhi M. Zhao Q. Wang L. Huang Z. Law J. (2022). Microengineered platforms for characterizing the contractile function of *in vitro* cardiac models. Microsystems and Nanoeng. 8 (1), 26. 10.1038/s41378-021-00344-0 PMC8882466 35299653

[B15] EMA (2022). ICH guideline E14/S7B: clinical and nonclinical evaluation of QT/QTc interval prolongation and proarrhythmic potential—Questions and answers—Scientific guideline.

[B16] Farhat A. Kennedy S. W. (2019). Adverse outcome pathway on aryl hydrogen receptor activation leading to early life stage mortality, via reduced VEGF OECD Series on adverse outcome pathways no. 16; OECD series on adverse outcome pathways. Paris: OECD Publishing. 10.1787/063e1bf4-en

[B17] FDA (2005). S7B nonclinical evaluation of the potential for delayed ventricular repolarization (QT interval prolongation) by human pharmaceuticals. 16237859

[B18] FDA (2021). S7A safety pharmacology studies for human pharmaceuticals. 12356097

[B19] Funahashi A. Matsuoka Y. Jouraku A. Morohashi M. Kikuchi N. Kitano H. (2008). CellDesigner 3.5: a versatile modeling tool for biochemical networks. Proc. IEEE 96 (8), 1254–1265. 10.1109/JPROC.2008.925458

[B20] Gadaleta D. Garcia De Lomana M. Serrano-Candelas E. Ortega-Vallbona R. Gozalbes R. Roncaglioni A. (2024). Quantitative structure–activity relationships of chemical bioactivity toward proteins associated with molecular initiating events of organ-specific toxicity. J. Cheminformatics 16 (1), 122. 10.1186/s13321-024-00917-x 39501321 PMC11539312

[B21] Gawron P. Hoksza D. Piñero J. Peña-Chilet M. Esteban-Medina M. Fernandez-Rueda J. L. (2023). Visualization of automatically combined disease maps and pathway diagrams for rare diseases. Front. Bioinforma. 3, 1101505. 10.3389/fbinf.2023.1101505 37502697 PMC10369067

[B22] Georgiadis N. Tsarouhas K. Dorne J.-L. C. M. Kass G. E. N. Laspa P. Toutouzas K. (2022). Cardiotoxicity of chemical substances: an emerging hazard class. J. Cardiovasc. Dev. Dis. 9 (7), 226. 10.3390/jcdd9070226 35877588 PMC9316944

[B23] Gisone I. Cecchettini A. Ceccherini E. Persiani E. Morales M. A. Vozzi F. (2022). Cardiac tissue engineering: multiple approaches and potential applications. Front. Bioeng. Biotechnol. 10, 980393. 10.3389/fbioe.2022.980393 36263357 PMC9574555

[B24] Global Burden of Cardiovascular Diseases and Risks 2023 Collaborators (2025). Global, regional, and national burden of cardiovascular diseases and risk factors in 204 countries and territories, 1990-2023. J. Am. Coll. Cardiol. 86 (22), 2167–2243. 10.1016/j.jacc.2025.08.015 40990886

[B25] Hoksza D. Gawron P. Ostaszewski M. Smula E. Schneider R. (2019). MINERVA API and plugins: opening molecular network analysis and visualization to the community. Bioinformatics 35 (21), 4496–4498. 10.1093/bioinformatics/btz286 31074494 PMC6821317

[B26] Hoksza D. Gawron P. Ostaszewski M. Hasenauer J. Schneider R. (2020). Closing the gap between formats for storing layout information in systems biology. Briefings Bioinforma. 21 (4), 1249–1260. 10.1093/bib/bbz067 31273380 PMC7373180

[B27] Jaylet T. Coustillet T. Smith N. M. Viviani B. Lindeman B. Vergauwen L. (2024). Comprehensive mapping of the AOP-wiki database: identifying biological and disease gaps. Front. Toxicol. 6, 1285768. 10.3389/ftox.2024.1285768 38523647 PMC10958381

[B28] Knapen D. Angrish M. M. Fortin M. C. Katsiadaki I. Leonard M. Margiotta‐Casaluci L. (2018). Adverse outcome pathway networks I: development and applications. Environ. Toxicol. Chem. 37 (6), 1723–1733. 10.1002/etc.4125 29488651 PMC6004608

[B29] Konala V. B. R. Kuhikar R. More S. Gossmann M. Lickiss B. Linder P. (2025). CiPA-qualified human iPSC-derived cardiomyocytes: a new frontier in toxicity testing by evaluating drug-induced arrhythmias. Toxicol. Vitro 108, 106100. 10.1016/j.tiv.2025.106100 40482844

[B30] Ladeira L. Verhoeven A. Van Ertvelde J. Jiang J. Gamba A. Sanz-Serrano J. (2025). Unlocking liver physiology: comprehensive pathway maps for mechanistic understanding. Front. Toxicol. 7, 1619651. 10.3389/ftox.2025.1619651 40692843 PMC12277266

[B31] Lekawanvijit S. Kompa A. R. Wang B. H. Kelly D. J. Krum H. (2012). Cardiorenal syndrome: the emerging role of protein-bound uremic toxins. Circulation Res. 111 (11), 1470–1483. 10.1161/CIRCRESAHA.112.278457 23139286

[B32] Lester R. M. (2021). Update on ICH E14/S7B cardiac safety regulations: the expanded role of preclinical assays and the “double‐negative” scenario. Clin. Pharmacol. Drug Dev. 10 (9), 964–973. 10.1002/cpdd.1003 34331518 PMC8456868

[B33] Lind L. Araujo J. A. Barchowsky A. Belcher S. Berridge B. R. Chiamvimonvat N. (2021). Key characteristics of cardiovascular toxicants. Environ. Health Perspect. 129 (9), 095001. 10.1289/EHP9321 34558968 PMC8462506

[B86] Maertens A. Kincaid B. Bridgeford E. Brochot C. Silva A. de C. (2025). From cellular perturbation to probabilistic risk assessments. ALTEX. 10.14573/altex.2501291 40418784

[B34] Mamoshina P. Rodriguez B. Bueno-Orovio A. (2021). Toward a broader view of mechanisms of drug cardiotoxicity. Cell. Rep. Med. 2 (3), 100216. 10.1016/j.xcrm.2021.100216 33763655 PMC7974548

[B35] Martens M. Meuleman A. B. Kearns J. De Windt C. Evelo C. T. Willighagen E. L. (2023). Molecular adverse outcome pathways: towards the implementation of transcriptomics data in risk assessments. bioRxiv. 10.1101/2023.03.02.530766

[B36] Mazein A. Ostaszewski M. Kuperstein I. Watterson S. Le Novère N. Lefaudeux D. (2018). Systems medicine disease maps: community-driven comprehensive representation of disease mechanisms. Npj Syst. Biol. Appl. 4 (1), 21. 10.1038/s41540-018-0059-y 29872544 PMC5984630

[B37] Mazein A. Lopata O. Reiche K. Sewald K. Alb M. Sakellariou C. (2025). An explorable model of an adverse outcome pathway of cytokine release syndrome related to the administration of immunomodulatory biotherapeutics and cellular therapies. Front. Immunol. 16, 1601670. 10.3389/fimmu.2025.1601670 40861455 PMC12371706

[B38] Mi H. Schreiber F. Moodie S. Czauderna T. Demir E. Haw R. (2015). Systems biology graphical notation: activity flow language level 1 version 1.2. 10.2390/BIECOLL-JIB-2015-265 26528563

[B39] Mortensen H. M. Martens M. Senn J. Levey T. Evelo C. T. Willighagen E. L. (2022). The AOP-DB RDF: applying FAIR principles to the semantic integration of AOP data using the research description framework. Front. Toxicol. 4, 803983. 10.3389/ftox.2022.803983 35295213 PMC8915825

[B40] Mortensen H. M. Gromelski M. Hench G. Martens M. Wittwehr C. Kumar S. (2025). The FAIR AOP roadmap for 2025: advancing findability, accessibility, interoperability, and re-usability of adverse outcome pathways. Comput. Toxicol. 35, 100368. 10.1016/j.comtox.2025.100368 40950714 PMC12425154

[B41] Murphy M. P. Bayir H. Belousov V. Chang C. J. Davies K. J. A. Davies M. J. (2022). Guidelines for measuring reactive oxygen species and oxidative damage in cells and *in vivo* . Nat. Metab. 4 (6), 651–662. 10.1038/s42255-022-00591-z 35760871 PMC9711940

[B42] Novère N. L. Hucka M. Mi H. Moodie S. Schreiber F. Sorokin A. (2009). The systems biology graphical notation. Nat. Biotechnol. 27 (8), 735–741. 10.1038/nbt.1558 19668183

[B43] OECD (2017). Guidance Document for the Use of Adverse Outcome Pathways in Developing Integrated Approaches to Testing and Assessment (IATA), OECD Series on Testing and Assessment. Paris: OECD Publishing, 260. 10.1787/44bb06c1-en

[B44] OECD. (2018). Users’ handbook supplement to the guidance document for developing and assessing adverse outcome pathways (OECD series on adverse outcome pathways no. 1; Paris: OECD Publishing). 1. 10.1787/5jlv1m9d1g32-en

[B45] OECD (2020). Overview of Concepts and Available Guidance related to Integrated Approaches to Testing and Assessment (IATA), OECD Series on Testing and Assessment. Paris: OECD Publishing, 329. 10.1787/cd920ca4-en

[B46] OECD (2025). The state of cardiovascular health in the european union. Paris: OECD Publishing. 10.1787/ea7a15f4-en

[B47] Ortega-Vallbona R. Palomino-Schätzlein M. Tolosa L. Benfenati E. Ecker G. F. Gozalbes R. (2024). Computational strategies for assessing adverse outcome pathways: hepatic steatosis as a case study. Int. J. Mol. Sci. 25 (20), 11154. 10.3390/ijms252011154 39456937 PMC11508863

[B48] Ozarde Y. Purandare D. Deshmukh S. Gadhave R. (2025). Heavy metals and cardiovascular health: uncovering links and health challenges. J. Trace Elem. Med. Biol. 89, 127648. 10.1016/j.jtemb.2025.127648 40228399

[B49] Paini A. Campia I. Cronin M. T. D. Asturiol D. Ceriani L. Exner T. E. (2022). Towards a qAOP framework for predictive toxicology—Linking data to decisions. Comput. Toxicol. 21, 100195. 10.1016/j.comtox.2021.100195 35211660 PMC8850654

[B50] Pan Z. Gong T. Liang P. (2024). Heavy metal exposure and cardiovascular disease. Circulation Res. 134 (9), 1160–1178. 10.1161/CIRCRESAHA.123.323617 38662861

[B51] Parish S. T. Aschner M. Casey W. Corvaro M. Embry M. R. Fitzpatrick S. (2020). An evaluation framework for new approach methodologies (NAMs) for human health safety assessment. Regul. Toxicol. Pharmacol. 112, 104592. 10.1016/j.yrtph.2020.104592 32017962

[B52] Perkins E. J. Ashauer R. Burgoon L. Conolly R. Landesmann B. Mackay C. (2019). Building and applying quantitative adverse outcome pathway models for chemical hazard and risk assessment. Environ. Toxicol. Chem. 38 (9), 1850–1865. 10.1002/etc.4505 31127958 PMC6771761

[B89] Posit team (2024). RStudio: Integrated Development Environment for R [R; PBC]. Posit Software. Available online at: https://posit.co/download/rstudio-desktop/ .

[B53] Proença S. Escher B. I. Fischer F. C. Fisher C. Grégoire S. Hewitt N. J. (2021). Effective exposure of chemicals in *in vitro* cell systems: a review of chemical distribution models. Toxicol. Vitro 73, 105133. 10.1016/j.tiv.2021.105133 33662518

[B88] R Core Team (2024). R: A Language and Environment for Statistical Computing. Available online at: https://www.R-project.org/ .

[B54] Qu Y. Li T. Liu Z. Li D. Tong W. (2023). DICTrank: the largest reference list of 1318 human drugs ranked by risk of drug-induced cardiotoxicity using FDA labeling. Drug Discov. Today 28 (11), 103770. 10.1016/j.drudis.2023.103770 37714406

[B55] Rangaswami J. Bhalla V. Blair J. E. A. Chang T. I. Costa S. Lentine K. L. (2019). Cardiorenal syndrome: classification, pathophysiology, diagnosis, and treatment strategies: a scientific statement from the American heart association. Circulation 139 (16), e840–e878. 10.1161/CIR.0000000000000664 30852913

[B56] Russom C. L. LaLone C. A. Villeneuve D. L. Ankley G. T. (2014). Development of an adverse outcome pathway for acetylcholinesterase inhibition leading to acute mortality. Environ. Toxicol. Chem. 33 (10), 2157–2169. 10.1002/etc.2662 24922588

[B57] Sang L. Zhou Z. Luo S. Zhang Y. Qian H. Zhou Y. (2024). An *in silico* platform to predict cardiotoxicity risk of anti-tumor drug combination with hiPSC-CMs based *in vitro* study. Pharm. Res. 41 (2), 247–262. 10.1007/s11095-023-03644-4 38148384 PMC10879352

[B58] Scardoni G. Tosadori G. Pratap S. Spoto F. Laudanna C. (2015). Finding the shortest path with PesCa: a tool for network reconstruction. F1000Research 4, 484. 10.12688/f1000research.6769.2 27781081 PMC5054806

[B59] Schaffert A. Murugadoss S. Mertens B. Paparella M. (2023). Cardiotoxicity of chemicals: current regulatory guidelines, knowledge gaps, and needs. ALTEX 40 (2), 337–340. 10.14573/altex.2301121 36648099

[B60] Schaffert A. Murugadoss S. Paparella M. Mertens B. (2024a). ALTERNATIVE deliverable D2.4 general structure of IATA for cardiotoxicity. 10.5281/zenodo.11109364

[B61] Schaffert A. Paparella M. Mertens B. (2024b). ALTERNATIVE deliverable D2.5 executive summary of the ALTERNATIVE project results, including questions for european regulators. 10.5281/zenodo.11401190

[B62] Schaffert A. Murugadoss S. Roos T. Linzalone N. Donzelli G. Gehring R. (2025). A data-driven approach for the development of a time-informed adverse outcome pathway-network for cardiotoxicity of environmental chemicals. bioRxiv. 10.64898/2025.12.22.695928

[B63] Serra A. Fratello M. Migliaccio G. Di Lieto E. Maia M. T. Saarimäki L. A. (2025). BMDx2: a tool for integrating toxicogenomics‐based dose‐dependency analysis and AOP‐Based mechanistic insights. Small Methods 9 (12), e01728. 10.1002/smtd.202501728 41229215 PMC12716184

[B64] Sewell F. Gellatly N. Beaumont M. Burden N. Currie R. De Haan L. (2018). The future trajectory of adverse outcome pathways: a commentary. Archives Toxicol. 92 (4), 1657–1661. 10.1007/s00204-018-2183-2 29549413 PMC5882624

[B65] Shankar P. Villeneuve D. L. (2023). AOP report: Aryl hydrocarbon receptor activation leads to early–life stage mortality *via* Sox9 repression-induced craniofacial and cardiac malformations. Environ. Toxicol. Chem. 42 (10), 2063–2077. 10.1002/etc.5699 37341548 PMC10772968

[B66] Shannon P. Markiel A. Ozier O. Baliga N. S. Wang J. T. Ramage D. (2003). Cytoscape: a software environment for integrated models of biomolecular interaction networks. Genome Res. 13 (11), 2498–2504. 10.1101/gr.1239303 14597658 PMC403769

[B67] Sharma E. Fotooh Abadi L. Kombe Kombe J. A. Kandala M. Parker J. Winicki N. (2025). Overview of methods that determine mitochondrial function in human disease. Metabolism 170, 156300. 10.1016/j.metabol.2025.156300 40389059 PMC12250752

[B68] Spinu N. Bal-Price A. Cronin M. T. D. Enoch S. J. Madden J. C. Worth A. P. (2019). Development and analysis of an adverse outcome pathway network for human neurotoxicity. Archives Toxicol. 93 (10), 2759–2772. 10.1007/s00204-019-02551-1 31444508

[B69] Spinu N. Cronin M. T. D. Enoch S. J. Madden J. C. Worth A. P. (2020). Quantitative adverse outcome pathway (qAOP) models for toxicity prediction. Archives Toxicol. 94 (5), 1497–1510. 10.1007/s00204-020-02774-7 32424443 PMC7261727

[B87] Sturla S. J. Boobis A. R. FitzGerald R. E. Hoeng J. Kavlock R. J. Schirmer K. (2014). Systems Toxicology: From Basic Research to Risk Assessment. Chem. Res. Toxicol. 27 (3), 314–329. 10.1021/tx400410s 24446777 PMC3964730

[B70] Staumont B. Ladeira L. Gamba A. Heusinkveld H. J. Piersma A. Masereeuw R. (2025). Mapping physiology: a systems biology approach for the development of alternative methods in toxicology. ALTEX 42, 301–307. 10.14573/altex.2412241 39918919

[B71] Tanabe S. Beaton D. Chauhan V. Choi I. Danielsen P. H. Delrue N. (2022). Report of the 1st and 2nd mystery of reactive oxygen species conferences. ALTEX 39, 336–338. 10.14573/altex.2203011 37470453

[B72] Tanabe S. Beaton D. Chauhan V. Choi I. Choi J. Clerbaux L.-A. (2023). Report of the 3rd and 4th mystery of reactive oxygen species conference. ALTEX 40, 689–693. 10.14573/altex.2307041 37889188

[B73] Van Ertvelde J. Verhoeven A. Maerten A. Cooreman A. Santos Rodrigues B. D. Sanz-Serrano J. (2023). Optimization of an adverse outcome pathway network on chemical-induced cholestasis using an artificial intelligence-assisted data collection and confidence level quantification approach. J. Biomed. Inf. 145, 104465. 10.1016/j.jbi.2023.104465 37541407

[B74] Varga Z. V. Ferdinandy P. Liaudet L. Pacher P. (2015). Drug-induced mitochondrial dysfunction and cardiotoxicity. Am. J. Physiology-Heart Circulatory Physiology 309 (9), H1453–H1467. 10.1152/ajpheart.00554.2015 26386112 PMC4666974

[B75] Verheijen M. Sarkans U. Wolski W. Jennen D. Caiment F. Kleinjans J. (2022). Multi-omics HeCaToS dataset of repeated dose toxicity for cardiotoxic and hepatotoxic compounds. Sci. Data 9 (1), 699. 10.1038/s41597-022-01825-1 36376331 PMC9663581

[B76] Verhoeven A. Van Ertvelde J. Boeckmans J. Gatzios A. Jover R. Lindeman B. (2024). A quantitative weight-of-evidence method for confidence assessment of adverse outcome pathway networks: a case study on chemical-induced liver steatosis. Toxicology 505, 153814. 10.1016/j.tox.2024.153814 38677583

[B77] Verhoeven A. Sanz-Serrano J. Vinken M. (2025). Mechanism-based new approach methodologies for *in vitro* detection of chemical-induced liver steatosis. Archives Toxicol. 100, 837–874. 10.1007/s00204-025-04235-5 41369776

[B78] Villeneuve D. L. Crump D. Garcia-Reyero N. Hecker M. Hutchinson T. H. LaLone C. A. (2014). Adverse outcome pathway (AOP) development I: strategies and principles. Toxicol. Sci. 142 (2), 312–320. 10.1093/toxsci/kfu199 25466378 PMC4318923

[B79] Villeneuve D. L. Angrish M. M. Fortin M. C. Katsiadaki I. Leonard M. Margiotta‐Casaluci L. (2018). Adverse outcome pathway networks II: network analytics. Environ. Toxicol. Chem. 37 (6), 1734–1748. 10.1002/etc.4124 29492998 PMC6010347

[B80] Vinken M. Knapen D. Vergauwen L. Hengstler J. G. Angrish M. Whelan M. (2017). Adverse outcome pathways: a concise introduction for toxicologists. Archives Toxicol. 91 (11), 3697–3707. 10.1007/s00204-017-2020-z 28660287 PMC5805086

[B81] Vuong N. Khilji S. Williams A. Adam N. Flores D. Fulton K. M. (2025). Integration of multi-omics and benchmark dose modeling to support adverse outcome pathways. Int. J. Radiat. Biol. 101 (3), 240–253. 10.1080/09553002.2024.2442694 39746153

[B82] Wickham H. (2016). Ggplot2. Springer International Publishing. 10.1007/978-3-319-24277-4

[B83] Wiklund L. Caccia S. Pípal M. Nymark P. Beronius A. (2023). Development of a data-driven approach to adverse outcome pathway network generation: a case study on the EATS-Modalities. Front. Toxicol. 5, 1183824. 10.3389/ftox.2023.1183824 37229356 PMC10203404

[B84] Wittwehr C. Clerbaux L.-A. Edwards S. Angrish M. Mortensen H. Carusi A. (2023). Why adverse outcome pathways need to be FAIR. ALTEX 41, 50–56. 10.14573/altex.2307131 37528748 PMC11177558

[B85] Wittwehr C. Audouze K. Burgdorf T. Clerbaux L.-A. Coerek E. Demuynck E. (2025). SKIG Report 2023-2024, Luxembourg: Publications Office of the European Union. Available online at: https://data.europa.eu/doi/10.2760/7749010,JRC140403

