# Supplementary material for: Cardiotoxicity adverse outcome pathway network: towards mechanistic and quantitative modelling
Source: Front Toxicol. 2026 May 18;8:1781536. doi: 10.3389/ftox.2026.1781536 (PMC13224945; doi:10.3389/ftox.2026.1781536)
Supplement: Supplementary file 1 [file Supplementaryfile1.zip › Supplementary_files/Supplementary_Table_1.docx]

Cardiotoxicity adverse outcome pathway network: towards mechanistic and quantitative modelling

**Authors:** Luiz Ladeira^1^, Devon A. Barnes^2^, Rosalinde Masereeuw^2^, Liesbet Geris^1, 3, 4§^, Bernard Staumont^1§^

**Affiliations**

1. Biomechanics Research Unit, GIGA Institute, University of Liège, Belgium;
2. Utrecht University, Division of Pharmacology, Utrecht Institute for Pharmaceutical Sciences, Utrecht, Netherlands;
3. Skeletal Biology and Engineering Research Center, KU Leuven, Belgium;
4. Biomechanics Section, Department of Mechanical Engineering, KU Leuven, Belgium.

**Supplementary Table 1.** Mapping of AOP key events to the key characteristics of cardiovascular toxicants.

| **Key Characteristic (KC)** |  | **Key Event (KE)** |
| --- | --- | --- |
| KC1: Impairs regulation of cardiac excitability |  | - Altered, Action Potential (KE698); - Blockade, L-Type Calcium Channels (KE1529); - Decrease, Calcium currents (KE1530); - Decreased, Sodium conductance 1 (KE585); - hERG channel blockade (KE2099); - Impaired, ion channels (KE697); - Increased, Atrioventricular block and bradycardia (KE444); - Increased, blood potassium concentration (KE1098); - Increased, cardiac arrhythmia (KE699, KE1106); - Inhibition of rapid delayed rectifying potassium current (KE2100); - Inhibition, sodium channel (KE584); - Prolongation of Action Potential (KE1961); - Prolongation of QT interval (KE1962); - Torsades de Pointes (KE1963). |
| KC2: Impairs cardiac contractility and relaxation |  | - Blockade, L-Type Calcium Channels (KE1529); - Decrease, Calcium binding to Troponin C (KE1531); - Decrease, Calcium currents (KE1530); - Decrease, Cardiac contractility (KE1532); - Disruption, Intracellular calcium mobilization (KE1536); - Decrease LV function (KE2215); - Decrease, Cardiac ejection fraction (KE1533). |
| KC3: Induces cardiomyocyte injury and death |  | - Cardiac hypertrophy (KE2001); - Cell injury/death (KE55); - Disruption, Sarcomere assembly (KE1537); - Increase, Cardiac remodelling (KE2084); - Increased, Cardiac fibrosis (KE1924); - Increased, Myocardial apoptosis (KE1918); - Ventricular remodeling (KE2002). |
| KC4: Induces proliferation of valve stroma |  | - Increased cellular proliferation and differentiation (KE1500). |
| KC5: Impacts endothelial and vascular function |  | - Endothelial cell dysfunction (KE1913, KE110); - Increased, Vascular smooth muscle cell activation (KE1925); - Reduced production, VEGF (KE948); - Vascular calcification (KE2000); - Vascular remodeling (KE2003). |
| KC6: Alters haemostasis |  | - Decreased, Prostaglandin F2alpha concentration, plasma (KE1104); - Inhibition, Cyclooxygenase 1 activity (KE1103). |
| KC7: Causes dyslipidaemia |  | - None |
| KC8: Impairs mitochondrial function |  | - Inhibition, ETC complexes of the respiratory chain (KE105); - Mitochondrial dysfunction (KE177, KE40). |
| KC9: Modifies autonomic nervous system activity |  | - ACh Synaptic Accumulation (KE10); - AchE Inhibition (KE12); - Activation, Nicotinic acetylcholine receptor (KE559); - Increased Cholinergic Signaling (KE39); - Increased, secretion of catecholamine (KE2004). |
| KC10: Induces oxidative stress |  | - Oxidative Stress (KE1392, KE1115). |
| KC11: Causes inflammation |  | - Activation, Inflammatory cytokines, chemokines, cytoprotective gene pathways (KE151); - Activation, Macrophages (KE1198); - Increase, COX-2 expression (KE1269). |
| KC12: Alters hormone signalling |  | - Activation, AhR (KE18); - Decrease, sox9 expression (KE2020); - Dimerization, AHR/ARNT (KE944); - Increase, slincR expression (KE2021); - Reduced dimerization, ARNT / HIF1-alpha (KE945). |
