# Supplementary figures and images for: Cardiotoxicity adverse outcome pathway network: towards mechanistic and quantitative modelling
Source: Front Toxicol. 2026 May 18;8:1781536. doi: 10.3389/ftox.2026.1781536 (PMC13224945; doi:10.3389/ftox.2026.1781536)

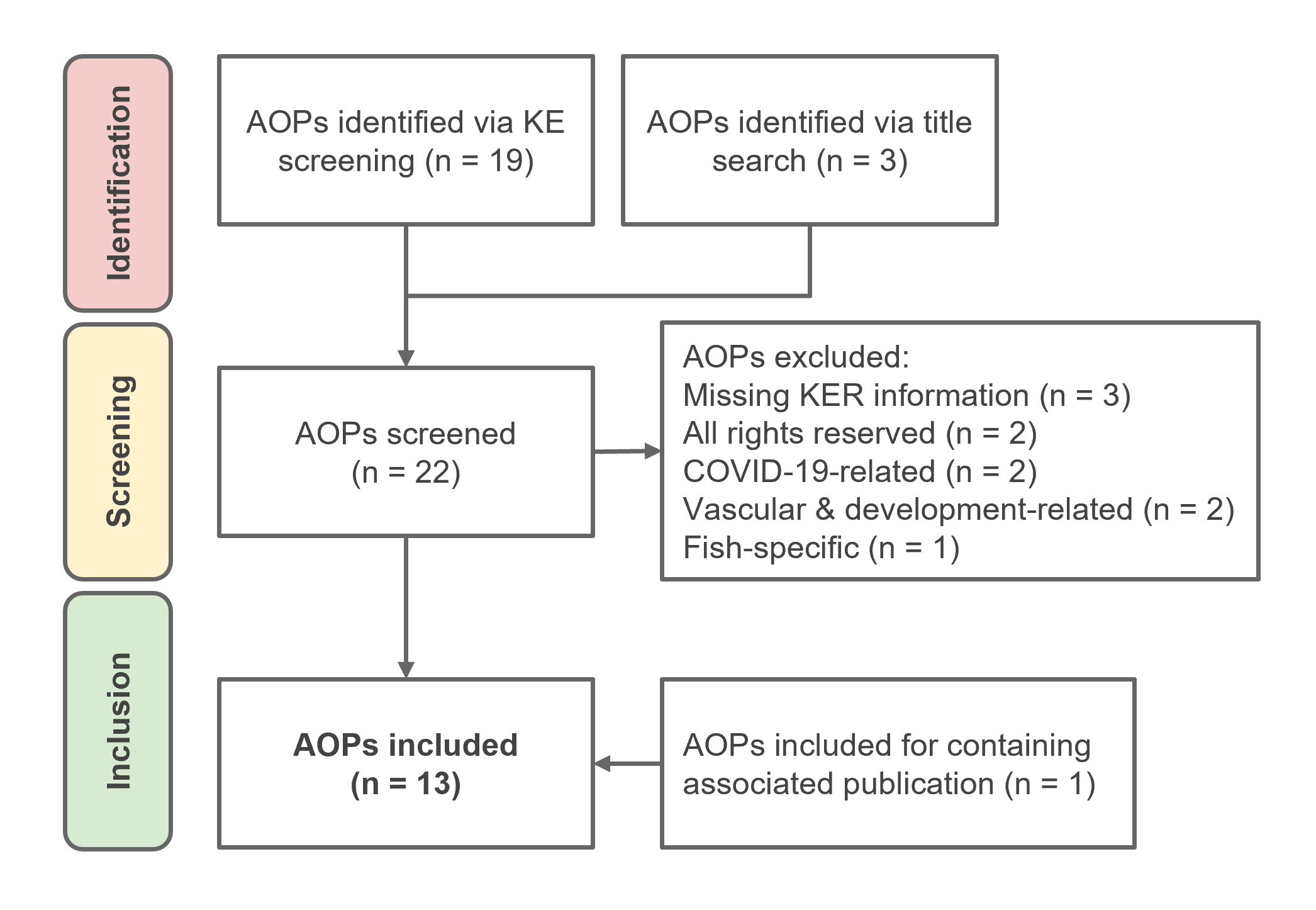

Supplement: Supplementary file 1 [file Supplementaryfile1.zip › Supplementary_files/Figures/Figure_1.png]

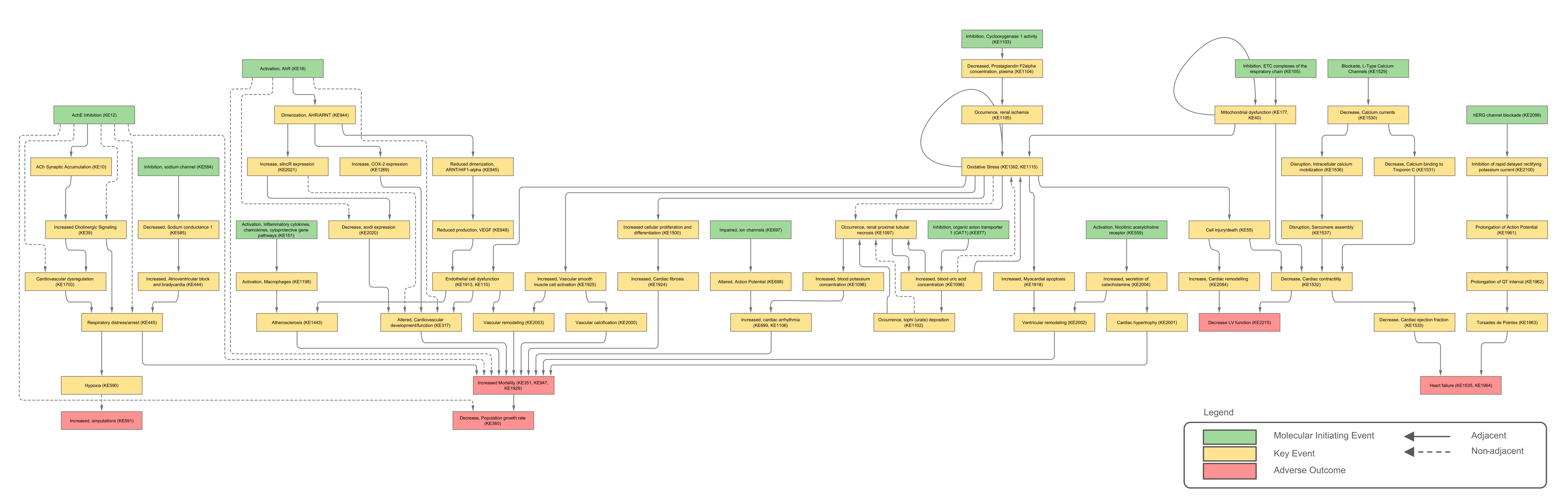

Supplement: Supplementary file 1 [file Supplementaryfile1.zip › Supplementary_files/Figures/Figure_2.png]

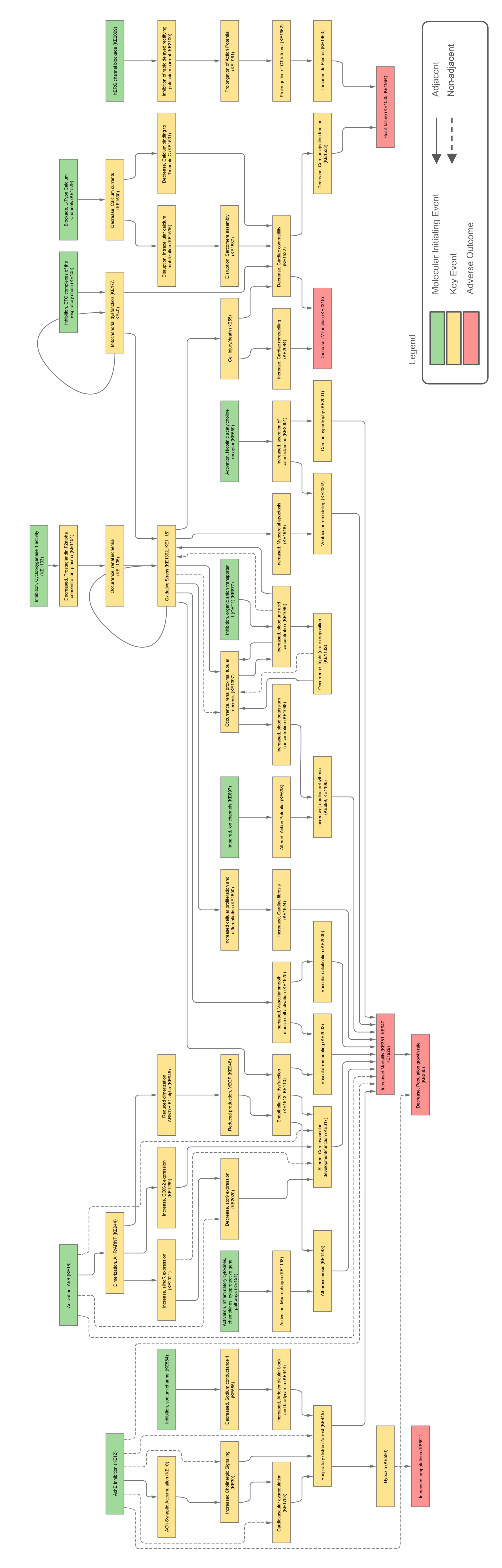

Supplement: Supplementary file 1 [file Supplementaryfile1.zip › Supplementary_files/Figures/Figure_2_vertical.png]

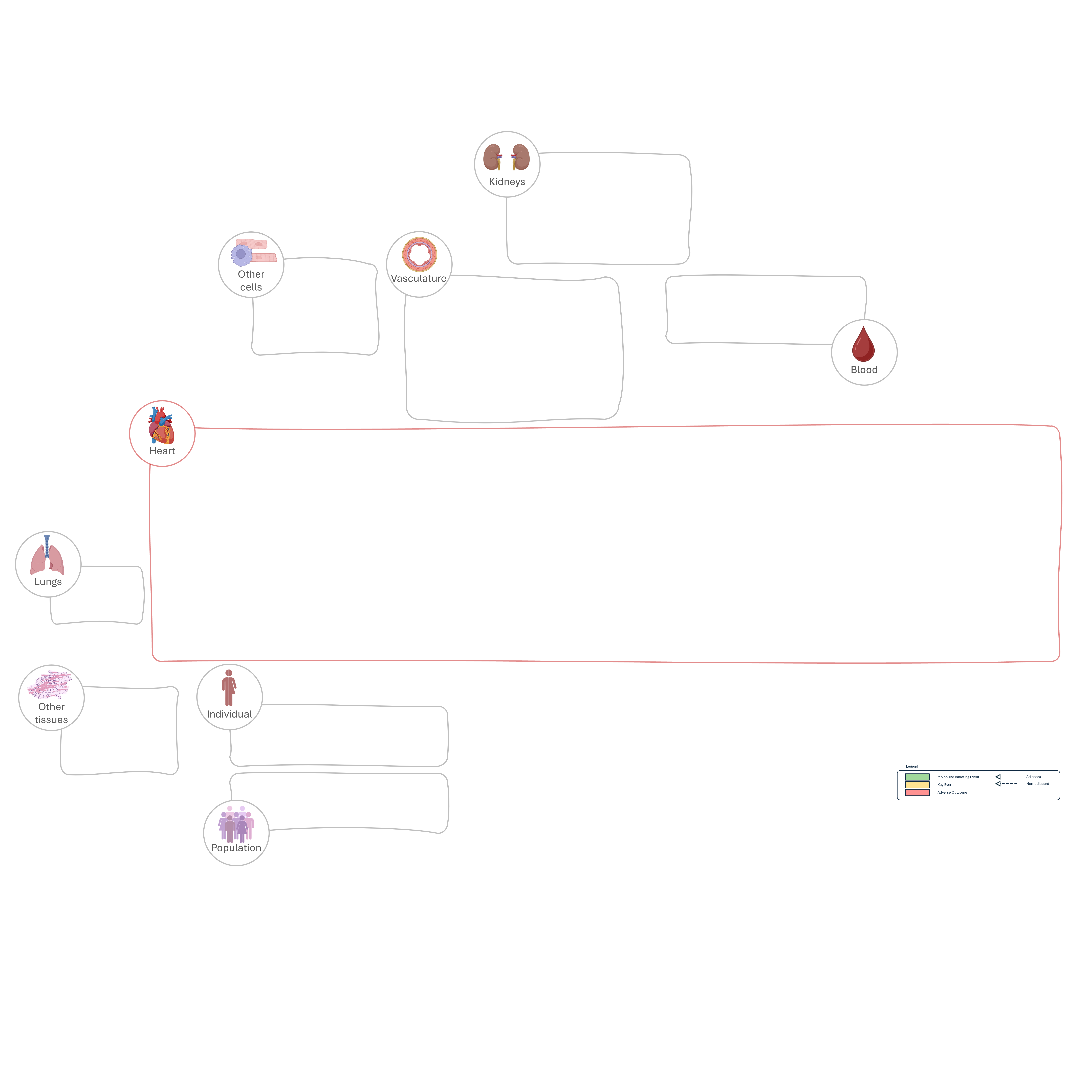

Supplement: Supplementary file 1 [file Supplementaryfile1.zip › Supplementary_files/network/glyphs/background.png]

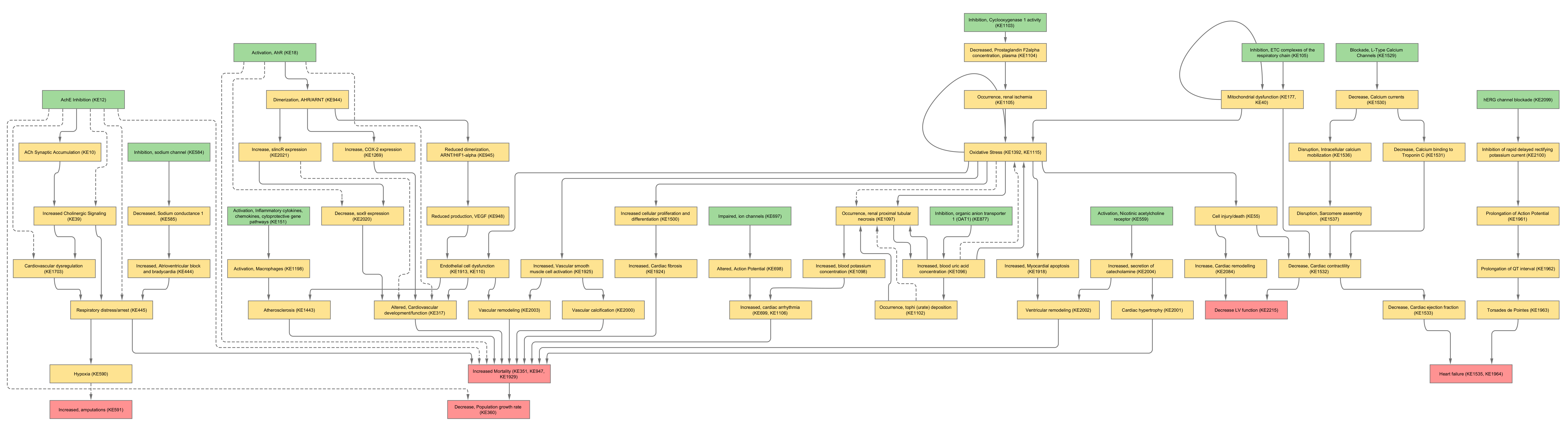

Supplement: Supplementary file 1 [file Supplementaryfile1.zip › Supplementary_files/network/network_cytoscape.png]

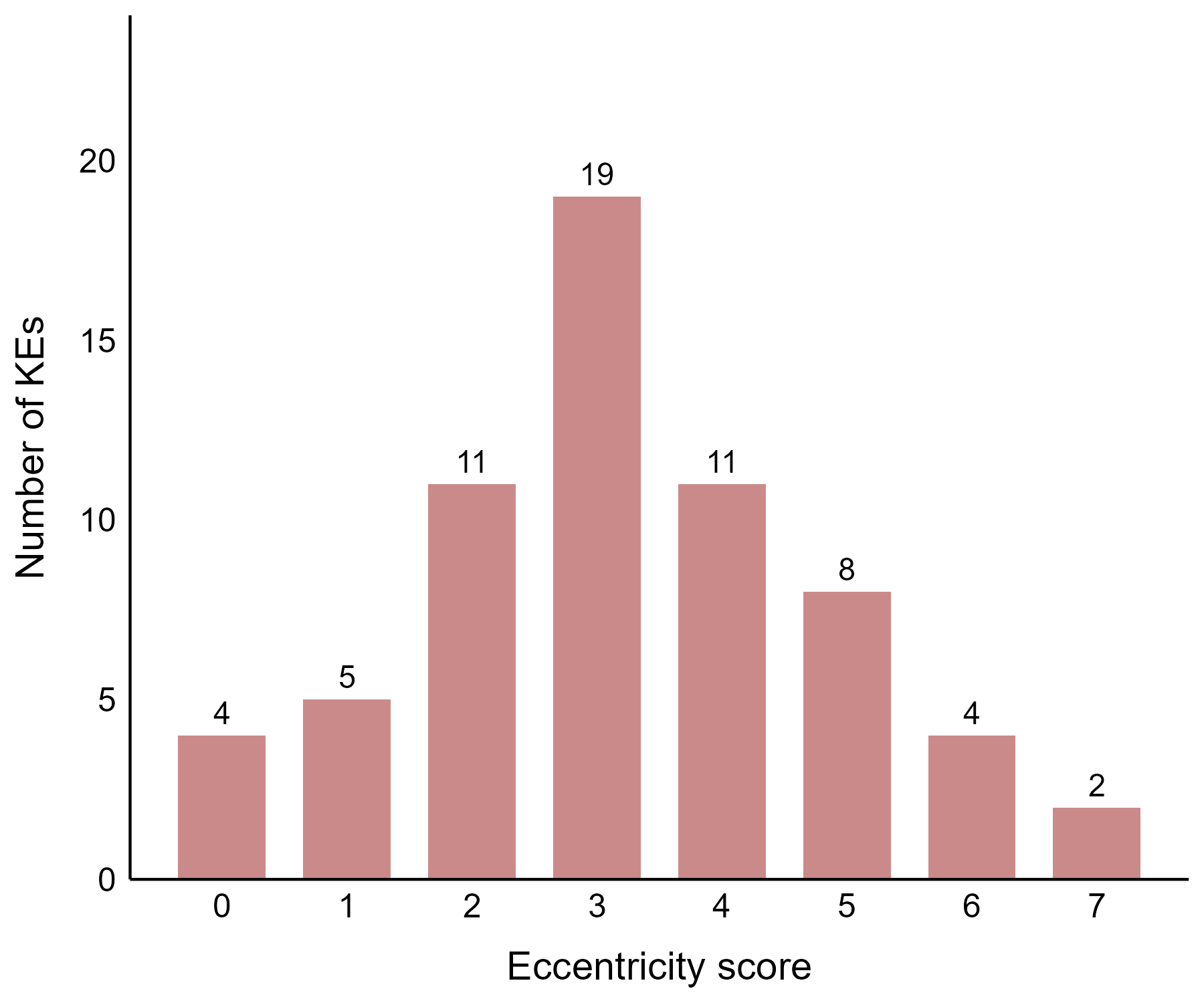

Supplement: Supplementary file 1 [file Supplementaryfile1.zip › Supplementary_files/outputs/figures/eccentricity_distribution.png]

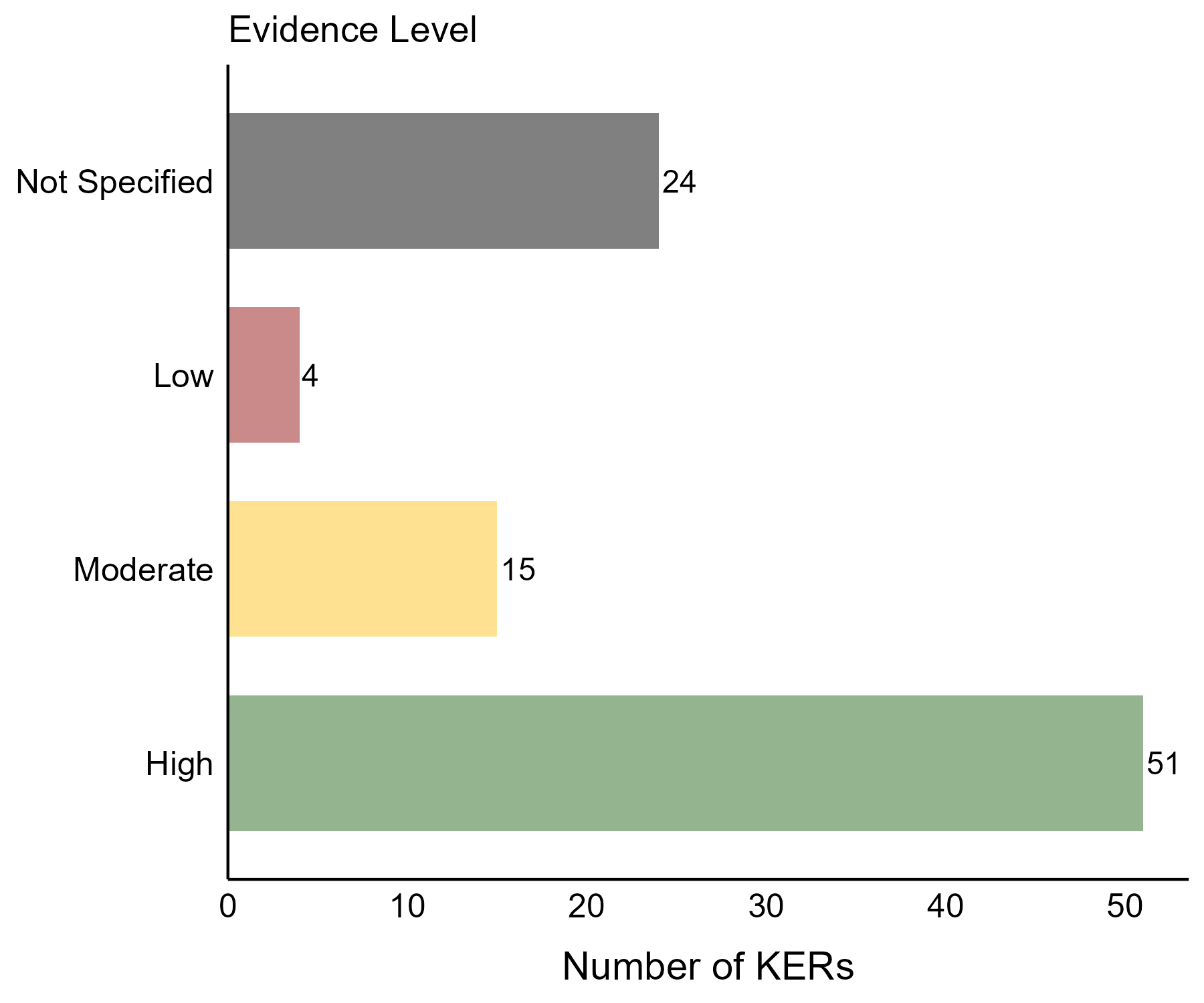

Supplement: Supplementary file 1 [file Supplementaryfile1.zip › Supplementary_files/outputs/figures/evidence_plot.png]

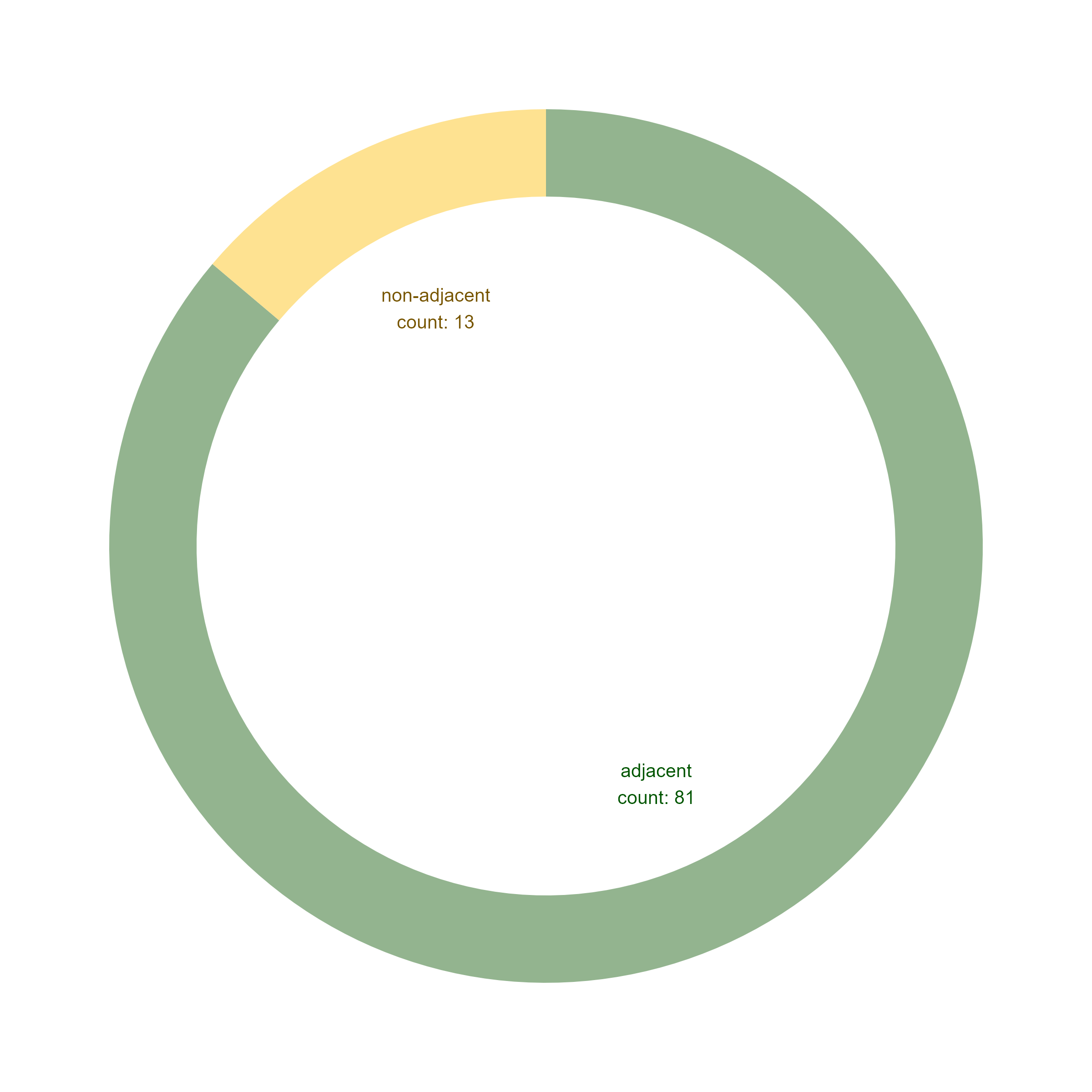

Supplement: Supplementary file 1 [file Supplementaryfile1.zip › Supplementary_files/outputs/figures/ke_adjacency_donut.png]

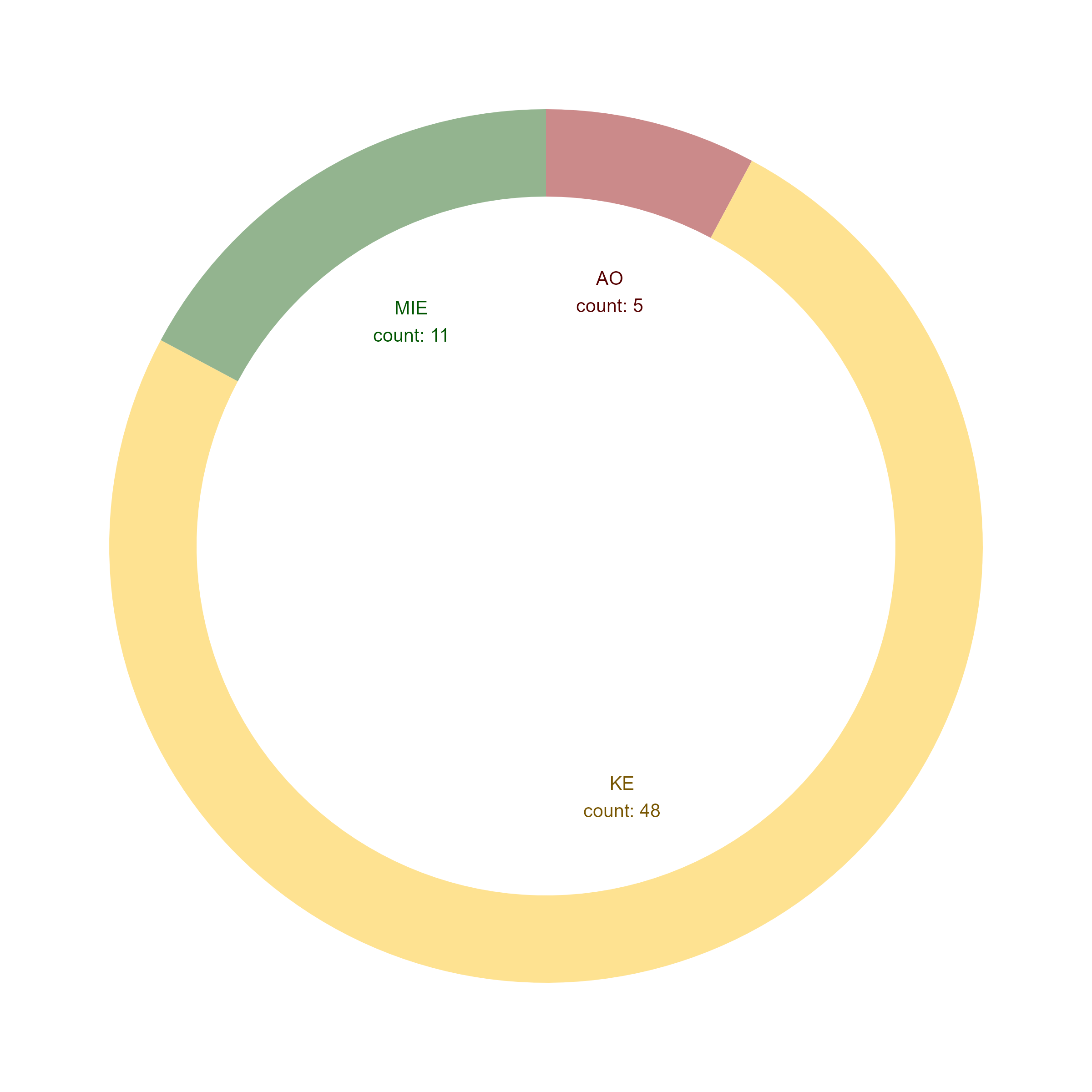

Supplement: Supplementary file 1 [file Supplementaryfile1.zip › Supplementary_files/outputs/figures/ke_distribution.png]

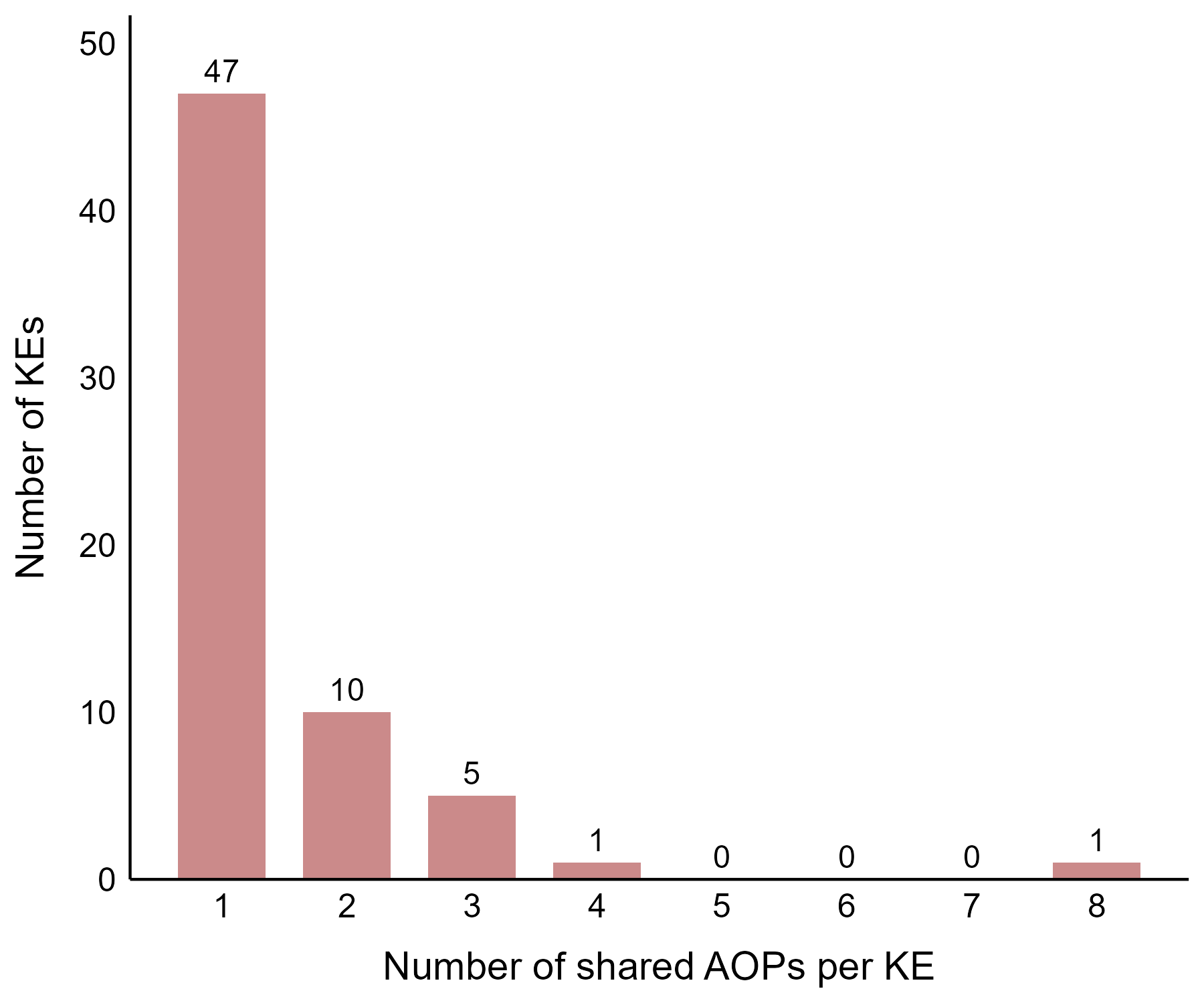

Supplement: Supplementary file 1 [file Supplementaryfile1.zip › Supplementary_files/outputs/figures/ke_shared_distribution.png]

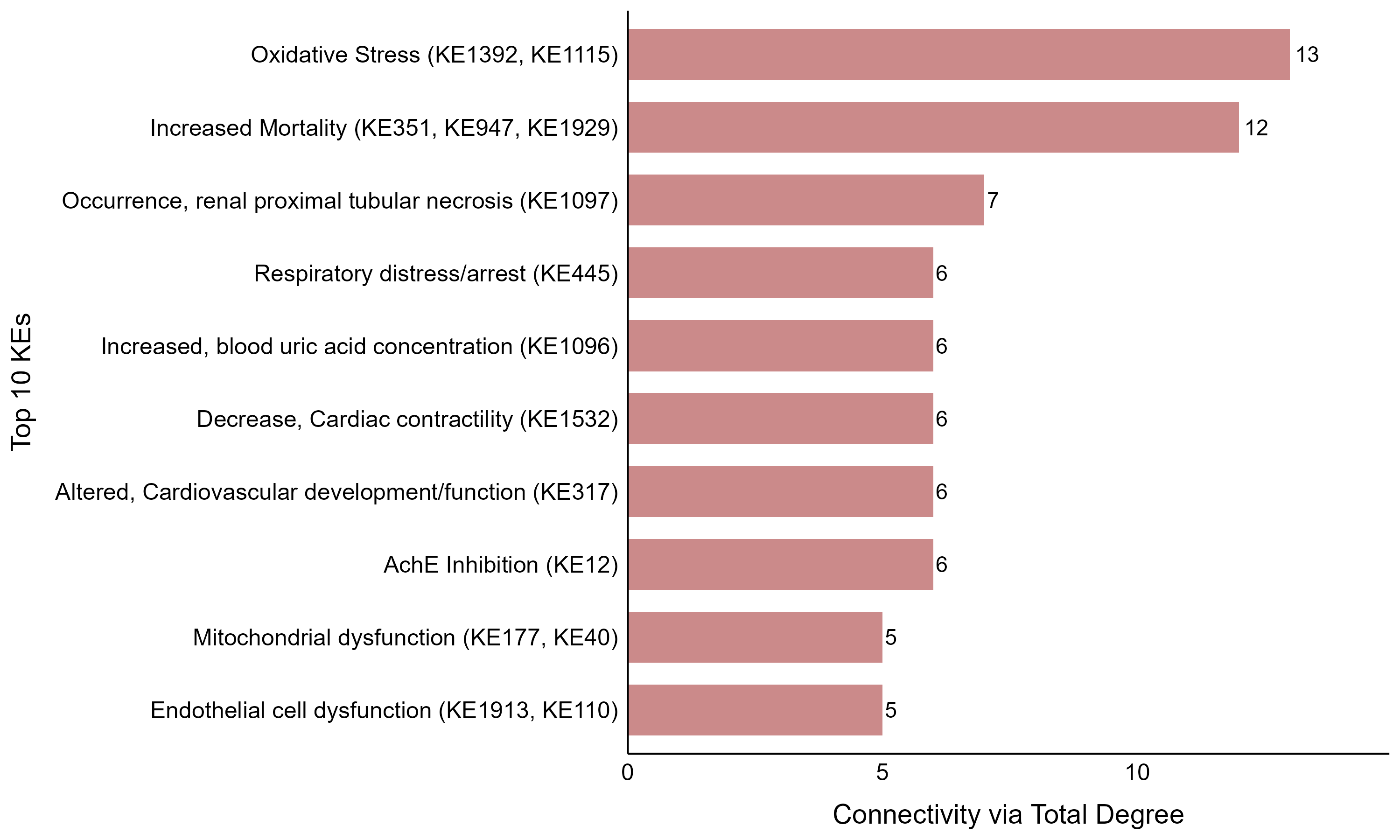

Supplement: Supplementary file 1 [file Supplementaryfile1.zip › Supplementary_files/outputs/figures/top_connectivity.png]

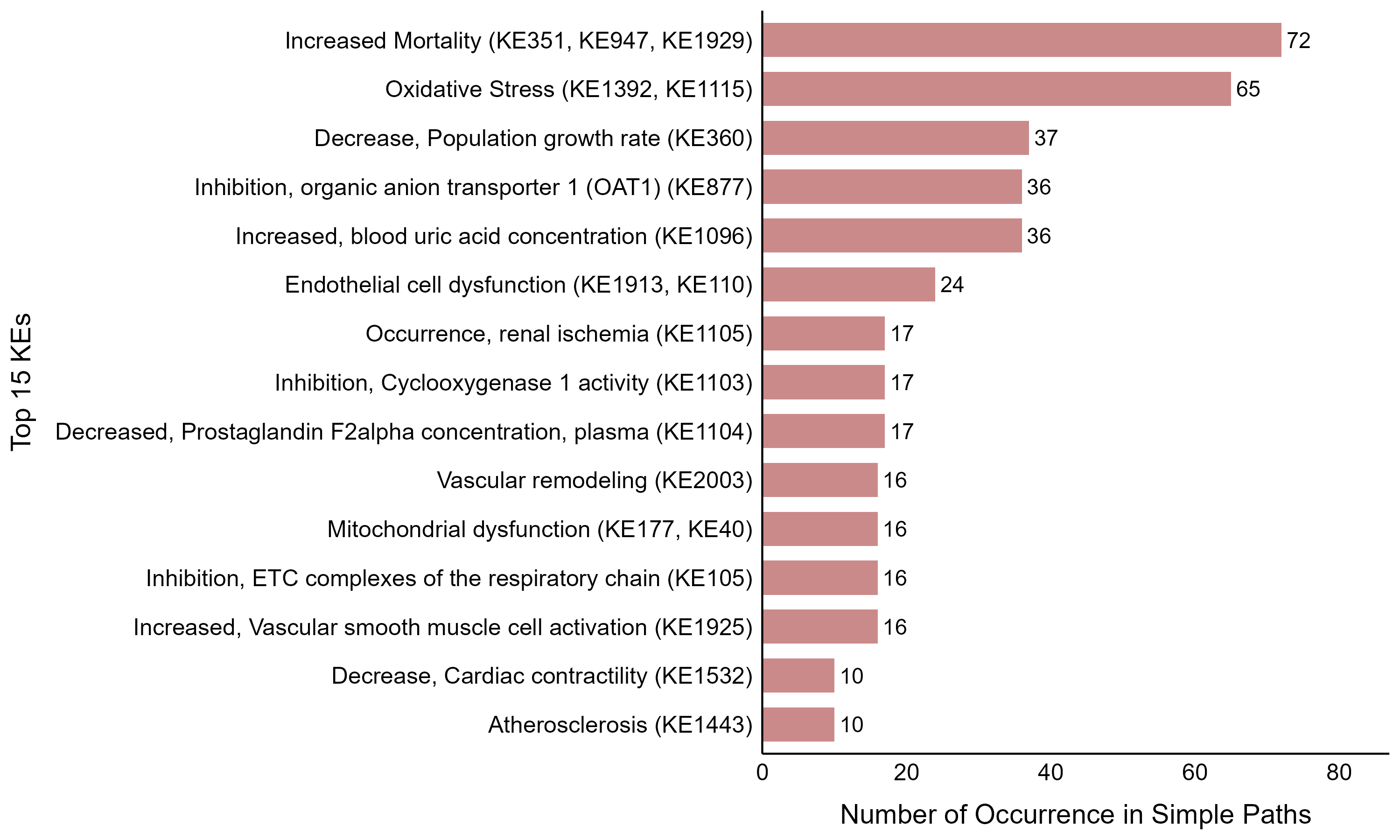

Supplement: Supplementary file 1 [file Supplementaryfile1.zip › Supplementary_files/outputs/figures/top_nodes_occurrence.png]

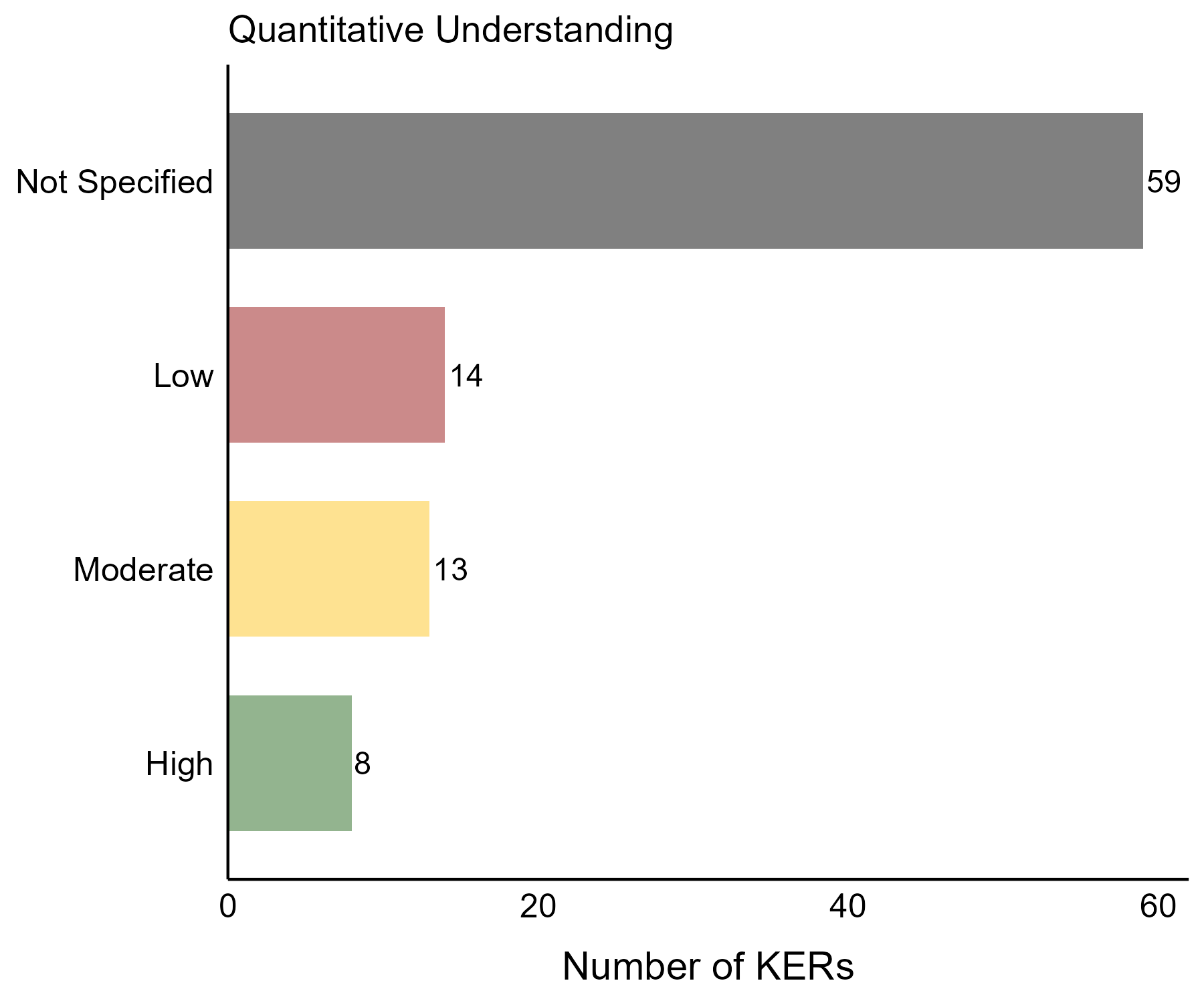

Supplement: Supplementary file 1 [file Supplementaryfile1.zip › Supplementary_files/outputs/figures/understanding_plot.png]
